# Disequilibrium between chloroplast proton motive force and ATP levels in *Arabidopsis*

**DOI:** 10.1038/s41477-026-02374-w

**Published:** 2026-08-17

**Authors:** Gustaf E. Degen, Markus Schwarzländer, Matthew P. Johnson

**Affiliations:** 1 Plants, Photosynthesis and Soil, School of Biosciences, University of Sheffield, Sheffield, UK; 2 Institute of Plant Biology and Biotechnology, University of Münster, Münster, Germany

**Keywords:** Plant sciences, Photosynthesis

## Abstract

Current dogma holds that CO_2_ fixation by photosynthesis requires additional ATP production via cyclic electron transfer, mediated by two distinct pathways involving the NADH dehydrogenase-like complex (NDH) and proton gradient regulation 5 protein (PGR5), to augment the ATP relative to NADPH produced by linear electron transfer. Here we use the MgATP^2−^ fluorescent biosensor ATeam1.03-nD/nA and find that, while proton motive force in the *Arabidopsis* *pgr5CAS* and *pgr5 ndho* mutants is only 50–75% that of the wild type, chloroplast MgATP^2−^ levels are unchanged. These data demonstrate that disequilibrium exists between the proton motive force and MgATP^2−^ concentration in vivo, indicating that cyclic electron transfer is not required for ATP augmentation.

## Abstract (web summary)

A fluorescent ATP biosensor reveals that chloroplast ATP levels remain unchanged in *Arabidopsis* mutants with a much weaker proton gradient, showing that cyclic electron transport protects photosynthesis rather than supplying extra ATP for CO_2_ fixation.

## Main

CO_2_ fixation in plants via the Calvin–Benson–Bassham (CBB) cycle is fuelled by conversion of light energy into chemical energy and reducing power in the form of ATP and NADPH. NADPH is formed via linear electron transfer (LET) from water to NADP^+^ and is coupled to movement of 6H^+^ across the thylakoid membrane via the water-splitting reaction at photosystem II (PSII) and Q-cycle at cytochrome *b*_6_*f* (cyt *b*_6_*f*). The transmembrane proton motive force (pmf) is harnessed by the chloroplast ATP synthase to generate ATP from ADP and phosphate (Pi). As the ATP synthase possesses 14 proton-binding c-subunits, the H^+^/ATP stoichiometry has been generally assumed to be 4.7 (14 divided by the 3 ATP molecules formed per 360° rotation of the enzyme)^1^. Therefore, 1.28 ATPs (6/4.7) can be formed per 1 NADPH^2^. However, the CBB cycle requires 9 ATP and 6 NADPH for CO_2_ fixation—a ratio of 1.5 (ref. ^3^). This is the central bioenergetic dogma underpinning oxygenic photosynthesis.

Any shortfall in ATP synthesis relative to NADPH production requires either the generation of additional pmf or the export of reductant from the chloroplast. In C3 angiosperms additional pmf can be generated via cyclic electron transfer (CET)^4^. CET involves the movement of electrons from the photosystem I (PSI) electron acceptor ferredoxin, which normally reduces NADP^+^, instead to the cyt *b*_6_*f* electron donor plastoquinone. This ensures that electrons pass through the Q-cycle a second time, thus increasing the H^+^/e^−^ stoichiometry. In C3 angiosperms, CET is mediated mainly by proton gradient regulation 5 (PGR5) and to a lesser extent by NADH dehydrogenase-like complex (NDH)^5^. *Arabidopsis thaliana* mutants lacking the NDH complex (*ndho*, *crr*) show only subtle phenotypes in, for example, low light, suggesting NDH’s role in pmf production can be largely compensated by increased PGR5-CET^5–8^. Mutants lacking PGR5 have been generated by ethyl methane sulfonate (*pgr5*) and CRISPR/CAS9-engineering (*pgr5CAS*), and these plants show substantially lower pmf^9,10^. Lower LET, decreased CO_2_-fixation rates and over-reduction of PSI electron acceptors are all consistent with a shortfall in ATP augmentation due to loss of pmf provided by CET^11,12^. Indeed, we previously interpreted the photosynthetic oscillations observed in *pgr5* during light transitions as evidence for a transient ATP shortfall arising from impaired CET^13^. However, whilst the aforementioned phenotypes have been measured using well-established gas-exchange and fluorescence techniques, direct evidence of a shortfall in ATP production in *pgr5* and *pgr5CAS* plants has never been provided in vivo.

An interesting and consistently observed feature of *pgr5* and *pgr5CAS* plants is their much higher proton conductivity (gH^+^) compared with the wild type (WT) at moderate-to-high light intensities^9^. High gH^+^ could indicate a lower phosphorylation potential in *pgr5* chloroplasts (less displacement of the ATP ⇌ ADP + Pi reaction from equilibrium) due to lower ATP concentrations^9^. Indeed, it has been suggested that ATP synthase in *pgr5* is ‘leaky’ (that is, it exhibits a non-productive proton leak), but this hypothesis was later ruled out through analysis of double mutants^14^. Alternatively, it was suggested that PGR5 might negatively regulate ATP synthase, restricting gH^+^ to enable pmf build-up, which would imply higher ATP levels in the *pgr5* mutant (a productive proton ‘leak’)^15^.

As CET generates additional pmf, of which the major component is the proton concentration gradient (∆pH) in chloroplasts^16^, it also plays a key role in triggering ∆pH-dependent photoprotective mechanisms. These include photosynthetic control (PCON), which controls the rate of electron transfer in the cyt *b*_6_*f* complex, preventing over-reduction and damage to PSI^17^, and non-photochemical quenching (NPQ), which protects PSII by dissipating excess excitation energy with its light harvesting antenna system^18^. Consistent with this concept, *pgr5* and *pgr5CAS* plants suffer from photoinhibition of PSI and a strong decrease in the level of NPQ^5,19^. Therefore, the loss of the additional pmf required for triggering photoprotective mechanisms on its own might explain the low LET and CO_2_ fixation phenotype in both *pgr5* mutants^20–22^.

Clearly, new approaches are required to distinguish between whether CET plays a key role in ATP augmentation, in photoprotection or both. Recently, a MgATP^2−^ fluorescent biosensor (ATeam1.03-nD/nA) was successfully expressed in *Arabidopsis* chloroplasts via the transketolase chloroplast transit peptide (TKTP) demonstrating how MgATP^2−^ levels change upon illumination due to LET activity^23,24^. The MgATP^2−^ biosensor relies on Forster resonance energy transfer (FRET) transfer between a monomeric super-enhanced cyan fluorescent protein (CFP) and circularly permuted monomeric Venus (a yellow fluorescent protein variant, YFP), which are both fused to an MgATP^2−^-sensing epsilon subunit of a bacterial ATP synthase^24^. The binding of ATP will bring both fluorophores into closer proximity increasing FRET efficiency, that is, upon excitation of CFP at 458 nm, energy will be transferred from CFP to YFP, resulting in an increased YFP:CFP emission ratio with increasing MgATP^2−^ concentration. Here, we establish the ATeam1.03-nD/nA fluorescent biosensor as a new tool in photosynthesis research to understand how MgATP^2−^ levels are correlated with pmf across the thylakoid membrane in WT and *pgr5CAS* plants.

**Fig. 1:**
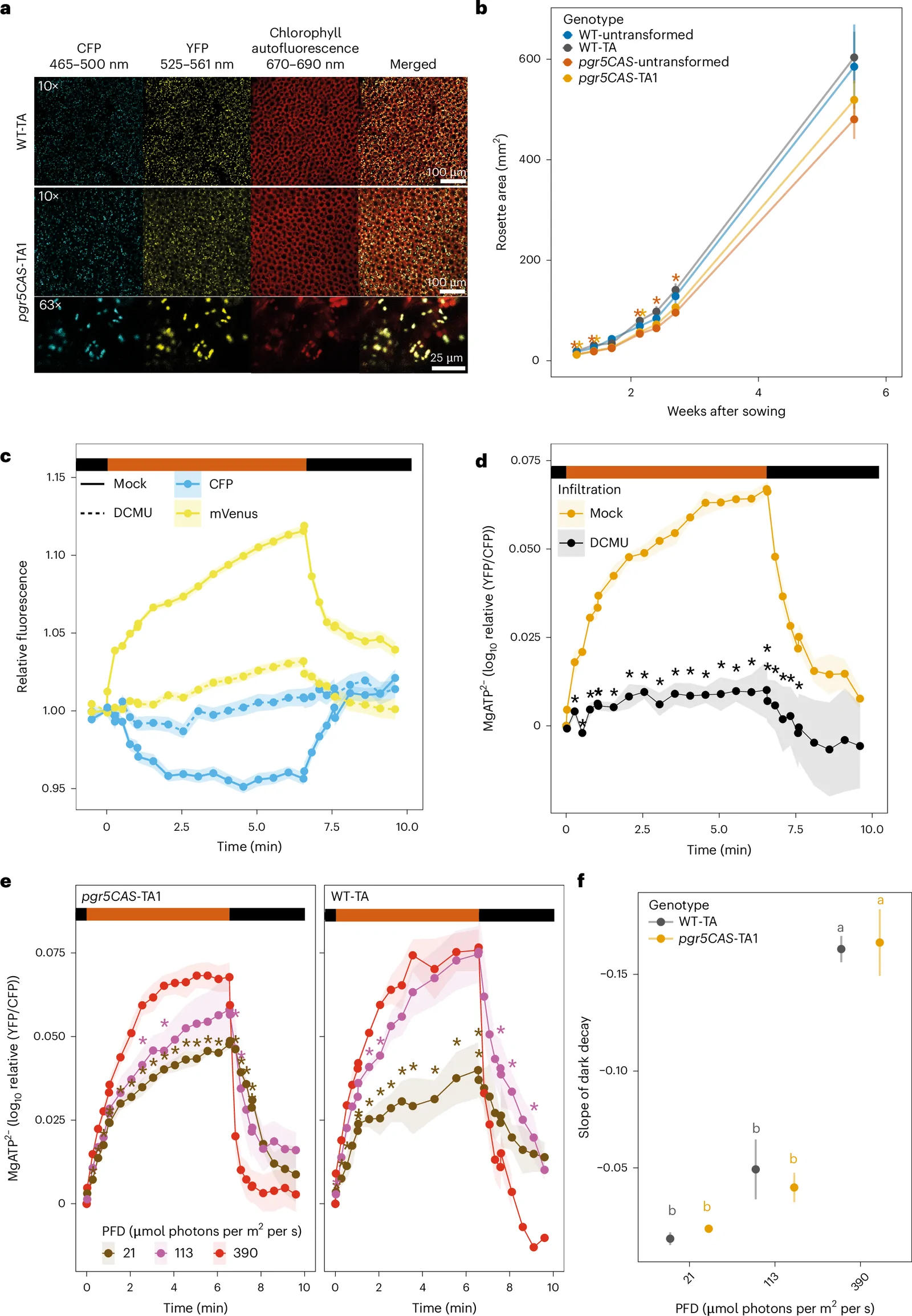
Light-dependent MgATP^2−^ accumulation in *pgr5CAS*-TA1. **a**, Representative confocal images of single plants of WT-TA and *pgr5CAS*-TA1 lines transformed with the chloroplast stroma-localized FRET MgATP^2−^ biosensor ATeam1.03-nD/nA. **b**, The rosette area of untransformed WT and *pgr5CAS* lines and transformed lines shown in **a** (*n* = 6, 6, 6 and 12). **c**, The intensities of the single CFP and YFP channels during dark–light–dark transitions, normalized to the last dark time-point (*t* = 0) of *pgr5CAS*-TA1 of leaves infiltrated with mock or 20 µM DCMU (*n* = 4). **d**, The MgATP^2−^ dynamics derived from normalized log_10_-transformed FRET ratios of mock and DCMU-treated *pgr5CAS*-TA1 leaves during dark–light–dark transitions (*n* = 4). **e**, MgATP^2−^ dynamics of *pgr5CAS*-TA1 and WT-TA leaves during 3 light intensities (*n* = 3). **f**, A comparison of the linear slope of the initial dark decay of MgATP^2−^ level following illumination (*n* = 3). For all panels, data points represent the mean of biological replicates (the *n* indicated above) and shaded areas/error bars represent s.e.m. Asterisks indicate significant differences between WT-untransformed and other genotypes in **a**, between mock and DCMU in **d** and between the photon flux density (PFD) of 390 and 113/21 in **e**. Letters indicate differences between light intensities in **f** calculated from a one-way anova followed by a Tukey HSD test; confidence level of 0.95. For **c** and **d**, the red bar corresponds to 390 µmol photons per m^2^ per s of red actinic light. For **e**, the red bar indicates when the light was turned on.

**Extended Data Fig. 1:**
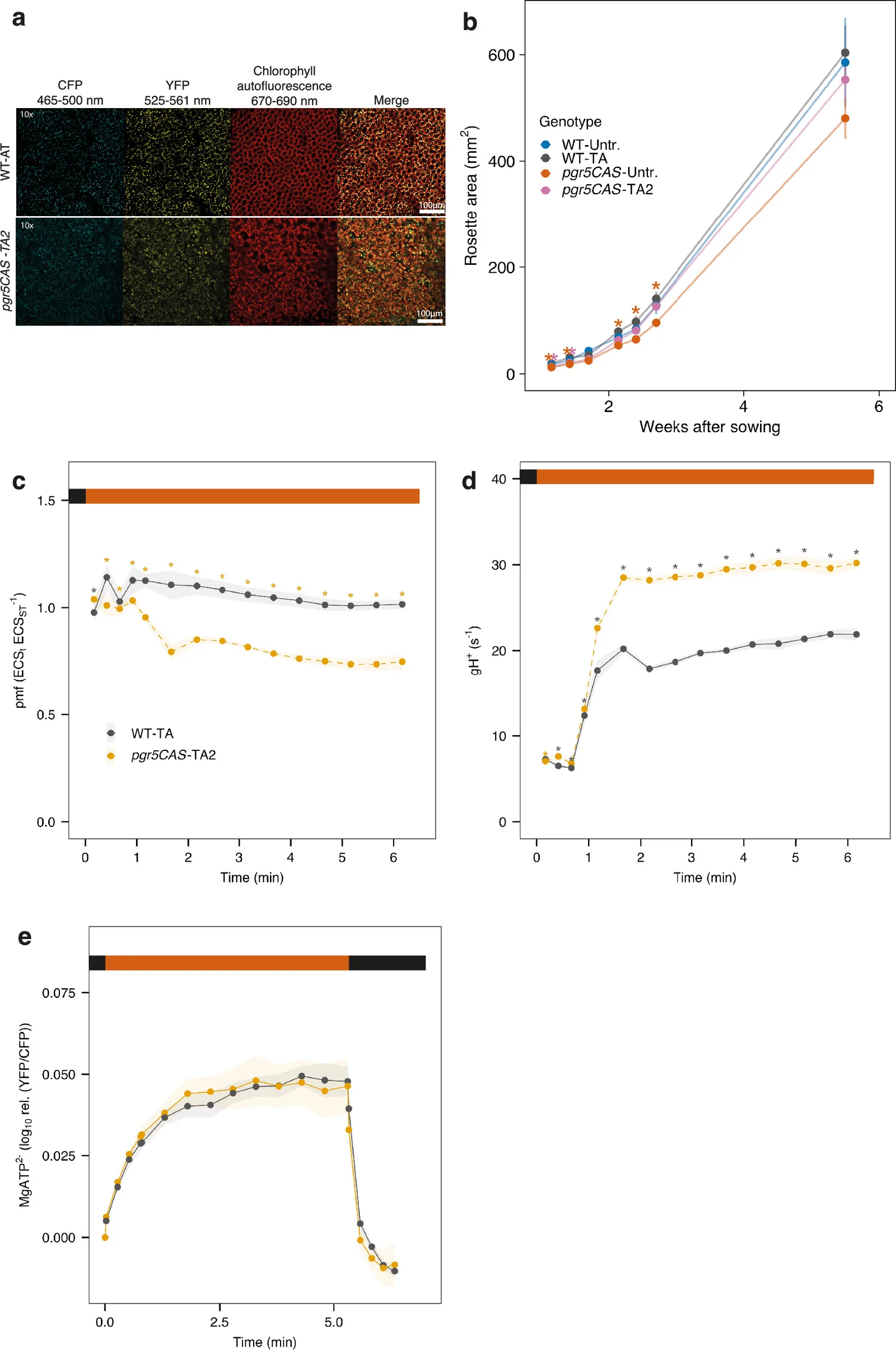
Supplementary Fig. 1: Validation of a second *pgr5CAS*-TA2. **a**) Confocal microscopy of WT-TA and *pgr5CAS*-TA2 showing the stable expression of the chloroplast stroma-localized FRET MgATP^2−^ biosensors ATeam1.03-nD/nA. **b**) Rosette area between untransformed and transformed WT and *pgr5CAS* lines, (n = 6, 6, 6, 12). **c**) pmf in WT-TA and *pgr5CAS*-TA2, (n = 3, 5). **d**) gH^+^ in WT-TA and *pgr5CAS*-TA2, (n = 3, 5). **e**) MgATP^2−^ levels in WT-TA and *pgr5CAS*-TA2, (n = 4, 3). For all panels, data points represent the mean of biological replicates (n indicated above) and shaded areas/error bars represent SEM. Asterisks indicate significant differences between WT-TA and *pgr5CAS-*TA2, calculated from a one-way anova followed by a Tukey HSD test, confidence level = 0.95.

In Fig. 1a, we show confocal fluorescence microscopy images of the TKTP-ATeam1.03-nD/nA biosensor in the WT background (hereafter WT-TA); as previously reported, the CFP and YFP fluorescence signals are co-located with the chlorophyll fluorescence, confirming their localization in the chloroplast^24^. We transformed the *pgr5CAS* mutant with the same construct to obtain two independent lines, *pgr5CAS* TKTP-ATeam 25 and 1513 (hereafter *pgr5CAS*-TA1 and *pgr5CAS*-TA2, respectively) (Fig. 1a and Extended Data Fig. 1). As with the WT, the YFP and CFP fluorescence signals co-locate with the chlorophyll autofluorescence of the chloroplast. We next performed growth assays to confirm that the transformed WT-TA, *pgr5CAS*-TA1 and *pgr5CAS*-TA2 plants did not show perturbed growth compared with the parent background strains (Fig. 1b and Extended Data Fig. 1). We used WT-TA and *pgr5CAS*-TA1 for all further experiments and confirmed that photosynthetic parameters were not significantly different compared with the respective background strains (Extended Data Fig. 4).

As confocal fluorescence microscopy measurements required detached leaves sandwiched between glass coverslips (limiting air contact) (Extended Data Fig. 3a), we sought to identify conditions yielding results comparable to those from attached leaves. We found that incubating leaves with 100 mM NaHCO_3_ (as a CO_2_ source) yielded results similar to those from attached leaves (Extended Data Fig. 3b–d); therefore, we used this concentration in all subsequent experiments. Previous work showed that the TKTP-ATeam1.03-nD/nA biosensor affinity (*K*_d_) for MgATP^2−^ decreases with increasing temperature; therefore, we rigorously controlled temperature, ensuring it did not exceed 22.5 °C even at the highest light intensity (Extended Data Fig. 3e). We then validated that the ATeam biosensor had sufficient dynamic range by infiltrating leaves with 0, 0.8, 1.6 and 3.2 mM equimolar ATP and MgCl_2_ in the presence of digitonin (Extended Data Fig. 3f), confirming that the sensor is well within the range of previously reported stromal ATP concentrations in the range of 0.5–1.8 mM (refs. ^23–26^). At this range of physiological ATP concentration, the ATeam1.03-nD/nA biosensor also displayed no pH sensitivity between pH 7.0 and 8.0 (refs. ^23,27^), which covers the previously measured stromal pH in the dark of ~7.2 and in the light of ~7.8 (refs. ^28–33^). The YFP variant (mVenus) used in the FRET sensor has a fluorescence p*K*a of ~6.0 (ref. ^23^), such that acid quenching of YFP emission only becomes significant below pH 6, well outside the physiological stromal range. The directionality of any residual pH sensitivity is nonetheless informative: the lower ΔpH characteristic of *pgr5CAS* would predict decreased light-driven stromal alkalinization and therefore a more acidic stromal pH than WT, which through acid quenching of mVenus would tend to decrease the apparent YFP:CFP ratio and underestimate MgATP^2−^. The WT-level MgATP^2−^ we observe in *pgr5CAS* therefore represents a conservative measurement and cannot arise from a pH-related sensor artefact.

We next confirmed that the ATeam1.03-nD/nA biosensor responded to light in the *pgr5CAS*-TA1 mutant (Fig. 1c). As expected, the CFP fluorescence decreased and the YFP fluorescence increased during a dark-to-light transition and then returned to close to the dark baseline upon cessation of illumination (Fig. 1c). This change was sensitive to 20 μM of the herbicide 3-(3,4-dichlorophenyl)-1,1-dimethylurea (DCMU) which poisons PSII and thus blocks LET (Fig. 1c). The ratio of the YFP:CFP fluorescence, indicative of the MgATP^2−^ level in the chloroplast, thus increased in the light and decreased again upon return to darkness (Fig. 1d), and this change was eliminated by DCMU. The MgATP^2−^ level in the *pgr5CAS*-TA1 mutant also showed a response which scaled with increasing light intensity, consistent with increased proton-coupled photosynthetic electron transfer (Fig. 1e). MgATP^2−^ levels decline more rapidly in the dark following higher intensity illumination, consistent with enhanced CO_2_ fixation rates resulting from increased CBB cycle activation (Fig. 1f). The rate of this post-illumination MgATP^2−^ decline, which reports CBB-driven ATP consumption once photophosphorylation ceases, was not significantly different between *pgr5CAS*-TA1 and WT-TA at matched light intensities in this saturating CO_2_ regime (100 mM NaHCO_3_) (Fig. 1e,f). This indicates that CBB-driven ATP demand is not appreciably altered in *pgr5CAS* despite its substantially lower pmf.

**Fig. 2:**
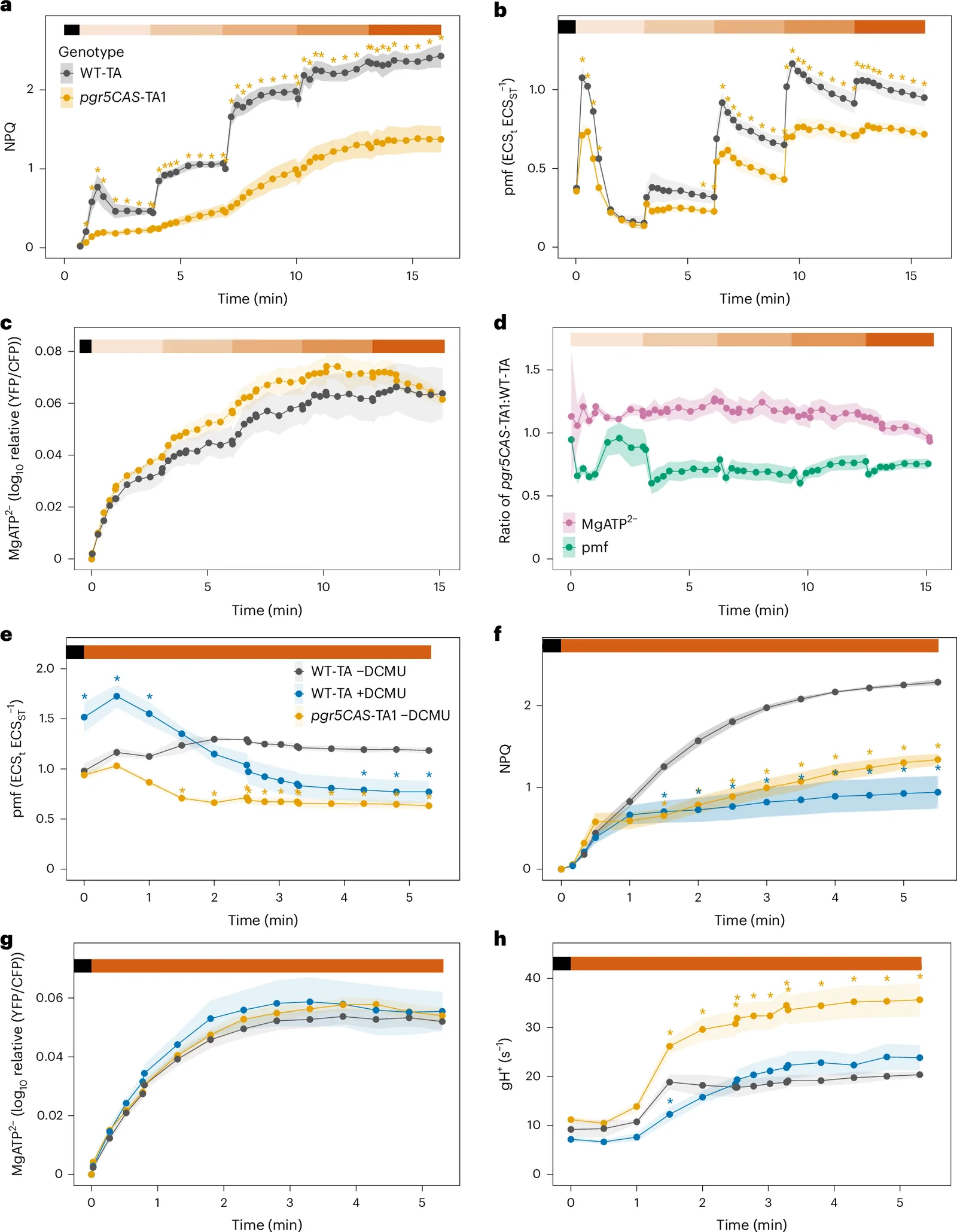
Photosynthetic parameters and MgATP^2−^ dynamics in WT-TA and *pgr5CAS*-TA1 plants. **a**, NPQ (*n* = 4). **b**, The pmf calculated as ECS_t_ ECS_ST_^−1^ (*n* = 4 and 7). **c**, The MgATP^2−^ dynamics derived from the chloroplast stroma-localized FRET MgATP^2−^ biosensor ATeam1.03-nD/nA. Log_10_-transformed FRET ratios relative to the last dark time-point (*t* = 0) before onset of illumination are shown (*n* = 5 and 4). **d**, The ratio of *pgr5CAS*-TA1 MgATP^2−^ and pmf levels relative to WT TKTP-ATeam at increasing light intensities derived from **b** and **c**. **e**, The pmf during dark-to-light transitions of WT TKTP-ATeam and *pgr5CAS*-TA1 infiltrated without or with 4 µM DCMU (*n* = 3, 5 and 3). **f**, The NPQ of the same plants and treatments as in **e** (*n* = 3, 4 and 3). **g**, The MgATP^2−^ dynamics of the same plants and treatments as in **e** (*n* = 11, 6 and 5). **h**, The proton conductance of the thylakoid membrane of the same plants and treatments as in **e** (*n* = 3, 5 and 3). For all panels, data points represent the mean of biological replicates (the *n* values indicated above, in the order of each legend) and shaded areas represent s.e.m. Asterisks indicate significant differences between WT-TA (−DCMU) and other genotypes or treatments, calculated from a one-way ANOVA followed by a Tukey HSD test; confidence level of 0.95. No statistics were performed for **d**. Coloured bars at the top of the panel indicate red actinic light intensities. For **a** (dual KLAS, peak *λ* = 622): 0, 59, 87, 163, 294 and 383 µmol photons per m^2^ per s. For **b** (dual PAM, peak *λ* = 622): 0, 50, 77, 152, 287 and 378 µmol photons per m^2^ per s. For **c** (LED ring combined with confocal microscope, peak *λ* = 644): 0, 54, 83, 178, 228 and 390 µmol photons per m^2^ per s. The light intensities during induction shown in **e**–**h** were 383, 378, 390 and 378 µmol photons per m^2^ per s, respectively.

**Extended Data Fig. 2:**
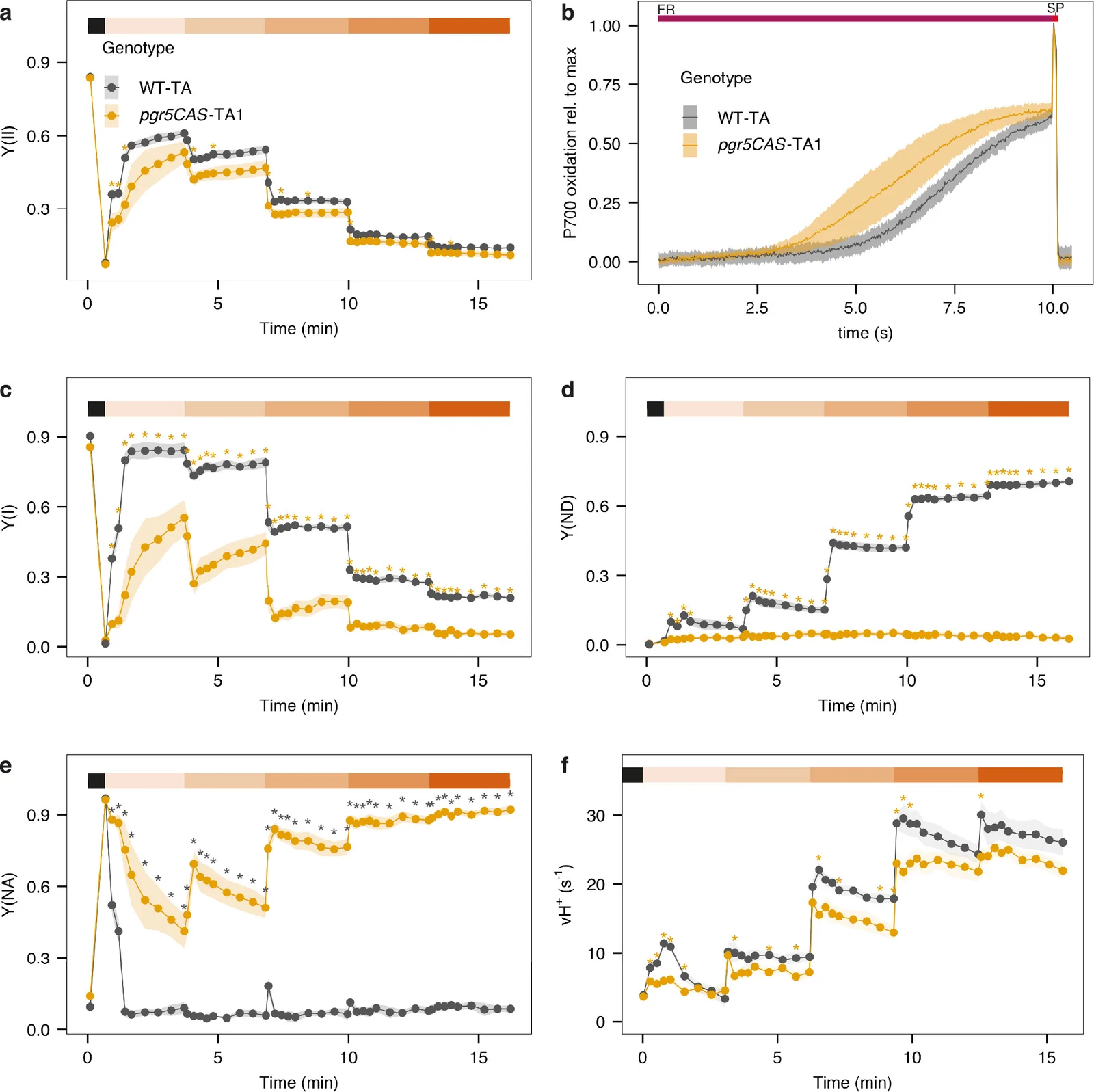
Supplementary Fig. 2: Photosynthetic parameters of WT TKTP-ATeam and *pgr5CAS*-TA1 in response to increasing light intensities. **a**) Yield of photosystem II (Y(II)), (n = 4). **b**) Oxidation of P700 under far red light normalized to a saturating flash at 10 s, (n = 4). **c**) Yield of photosystem I (Y(I)), (n = 4) **d**) Donor-side limitation of PSI, also referred to as P700 oxidation (Y(ND)), (n = 4) **e**) Acceptor-side limitation of PSI, also referred to as P700 reduction (Y(NA)), (n = 4). **f**) Proton flux through the thylakoid membrane, (n = 4, 7). For all panels, data points represent the mean of biological replicates (n indicated above) and shaded areas represent SEM. Asterisks indicate significant differences between WT-TA and other genotypes, calculated from a one-way anova followed by a Tukey HSD test, confidence level = 0.95. Coloured bars at the top of the panel indicate red actinic light intensities. The same light intensities as in Fig. 2a–d were used.

Subsequently, we confirmed the photosynthetic phenotype of the *pgr5CAS*-TA1 plants was consistent with the previously reported phenotype for the *pgr5CAS* mutant using chlorophyll fluorescence, P700 and electrochromic shift (ECS) absorption measurements at light intensities of 0, 54, 83, 178, 228 and 390 µmol photons per m^2^ per s. Compared with the WT-TA, the *pgr5CAS*-TA1 plants showed decreased NPQ (Fig. 2a) and lower PSII quantum yield (Y(II)) (Extended Data Fig. 2a) with increasing light intensity. Similarly, *pgr5CAS*-TA1 showed faster P700 oxidation under far-red light, indicative of lower CET (Extended Data Fig. 2b), and under red light showed a lower PSI quantum yield (Extended Data Fig. 2c), decreased PSI donor side limitation (Extended Data Fig. 2d) and increased PSI acceptor-side limitation (Extended Data Fig. 2e). Consistent with this phenotype, the *pgr5CAS*-TA1 plants showed lower pmf with increasing light intensity compared with WT-TA (Fig. 2b), mostly as a result of lower proton flux (vH^+^) consistent with a loss in CET (Extended Data Fig. 2f).

Despite these differences in key photosynthetic parameters and pmf, we found the *pgr5CAS*-TA1 plants showed very similar chloroplast MgATP^2−^ levels compared with the WT-TA (Fig. 2c). In both cases the increasing light intensity led to an increase in MgATP^2−^ levels with saturation reached at 228 µmol photons per m^2^ per s for both sets of plants. The disparity between pmf and MgATP^2−^ levels is shown in Fig. 2d, where *pgr5CAS*-TA1 plants maintain ~100% of the WT ATP level with only ~70–75% of the pmf.

Our biosensor data reveal significant disequilibrium between thylakoid pmf and chloroplast MgATP^2−^ levels in vivo. We tested this further by infiltrating WT-TA leaves with subsaturating 4 μM DCMU. Partial inhibition of photosynthetic electron transfer lowered pmf in the WT by ~50%, to a level consistent with that observed in *pgr5CAS*-TA1 after 5 min illumination at 390 µmol photons per m^2^ per s (Fig. 2e). The lower pmf in the 4 μM DCMU-treated WT-TA leaves also lowered NPQ to a level commensurate with that in *pgr5CAS*-TA1 (Fig. 2f). Nonetheless, despite the 50% drop in pmf, ATP levels in the 4 μM DCMU-treated WT-TA leaves were not significantly different from those in untreated WT-TA or *pgr5CAS*-TA1 (Fig. 2g). Interestingly, despite a significantly higher gH^+^ in *pgr5CAS*-TA1 compared with the WT-TA, the MgATP^2−^ levels remain similar (Fig. 2h). In the independent *pgr5CAS*-TA2 line, we also observed a significantly decreased pmf and increased gH^+^ but no differences in MgATP^2−^ levels compared with WT-TA (Extended Data Fig. 1c–e).

We considered the possibility that chloroplast MgATP^2−^ levels in *pgr5CAS*-TA1 plants were augmented by increased mitochondrial respiratory electron transfer and ATP import as observed in *Chlamydomonas*^34^. To test this further, we infiltrated plants with the specific inhibitor of the mitochondrial ATP synthase, oligomycin (Extended Data Fig. 5). Crucially, no differences were observed in chloroplast MgATP^2−^ levels between WT-TA and *pgr5CAS*-TA1 plants in the presence of oligomycin, showing that no augmentation of chloroplast ATP levels by mitochondria occurs in *Arabidopsis*. Indeed, infiltration rather increased chloroplast MgATP^2−^ levels in both WT-TA and *pgr5CAS*-TA1 plants (Extended Data Fig. 5). This finding is consistent with previous demonstrations that cytosolic ATP cannot readily enter chloroplasts of photosynthetically active tissues^24^, and with the observation that *pgr5* mutants preferentially upregulate the non-proton-pumping mitochondrial alternative oxidase (AOX) rather than the proton-pumping complex III pathway^35,36^, a metabolic shift that disposes of chloroplast-derived reductant exported via the malate valve without compensatory mitochondrial ATP production.

**Fig. 3:**
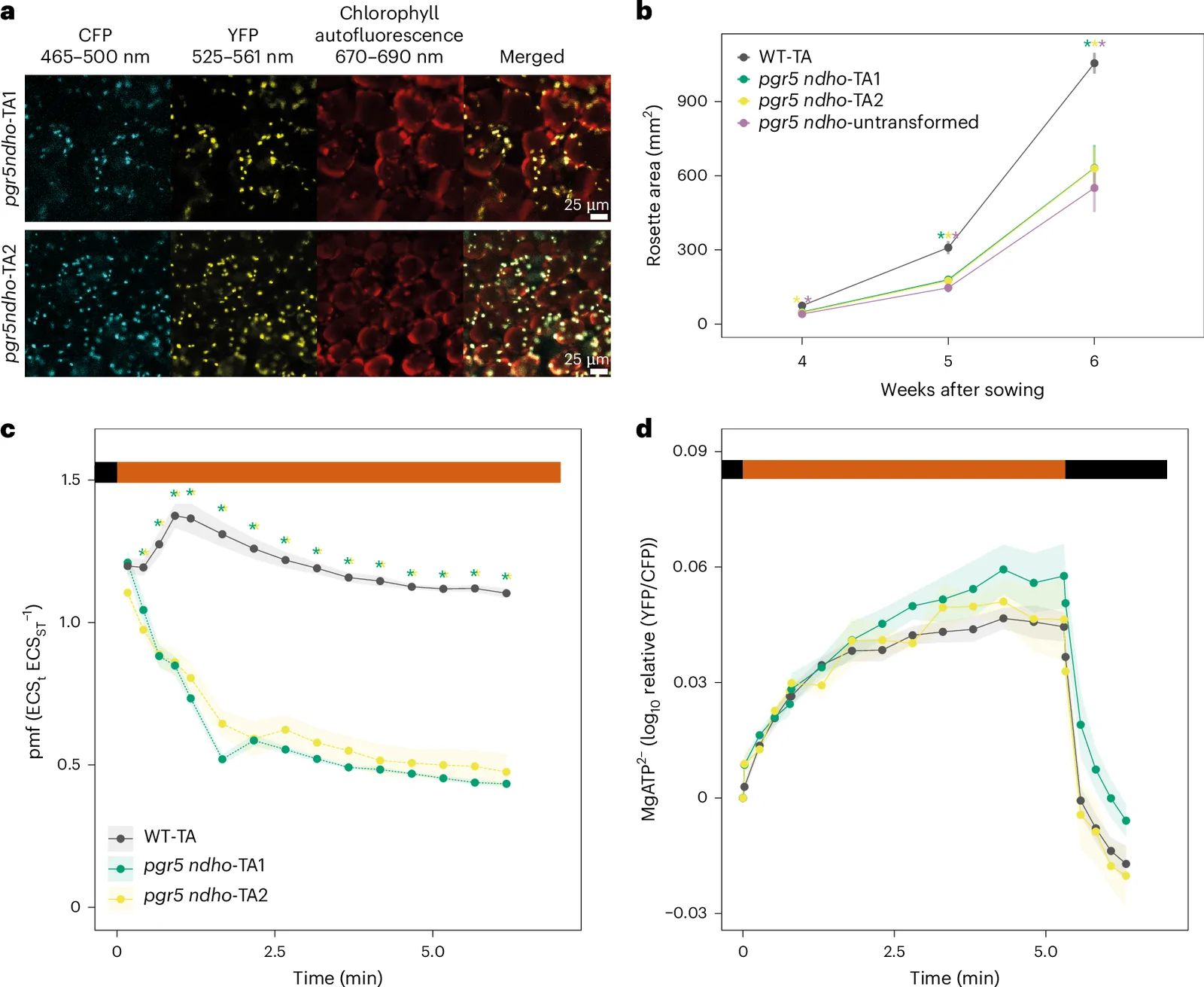
Light-dependent MgATP^2−^ accumulation in *pgr5 ndho*-TA. **a**, Representative confocal images of single plants of *pgr5ndho*-TA1 and *pgr5ndho*-TA2 lines transformed with the chloroplast stroma-localized FRET MgATP^2−^ biosensor ATeam1.03-nD/nA. **b**, The rosette area of untransformed WT and *pgr5 ndho* lines and transformed lines shown in **a** (*n* = 4). **c**, The pmf calculated as ECS_t_ ECS_ST_^−1^ during photosynthetic induction at 378 µmol photons per m^2^ per s (*n* = 3, 4 and 4). **d**, MgATP^2−^ dynamics during photosynthetic induction at 390 µmol photons per m^2^ per s (*n* = 6, 3 and 3). For all panels, data points represent the mean of biological replicates (the *n* values indicated above, in the order of the legend) and shaded areas/error bars represent s.e.m. Asterisks indicate significant differences between WT-untransformed and other genotypes in **a**, and between WT-TA and the two *pgr5 ndho* lines in **c** and **d**, calculated from a one-way ANOVA followed by a Tukey HSD test; confidence level of 0.95.

Previous work has shown that the growth phenotype of *pgr5* plants was significantly worsened in a double mutant also lacking the NDH pathway, which could indicate some redundancy between the two pathways in ATP augmentation^5,6^. To test this idea further we transformed the *pgr5 ndho* double mutant with the ATeam1.03-nD/nA biosensor, obtaining two independent lines: *pgr5 ndho*-TA1 and *pgr5 ndho-*TA2 (Fig. 3a). The growth of these lines was similar to *pgr5 ndho* and significantly slower than the WT (Fig. 3b), and photosynthetic parameters were identical to the background strain (Extended Data Fig. 4). Consistent with our DCMU titration above, although pmf was decreased to just 50% of the WT level in *pgr5 ndho*-TA1/2 (Fig. 3c), the chloroplast MgATP^2−^ levels were not significantly different to the WT-TA.

Overall, our results overturn two decades of dogma in photosynthesis research that CET is necessary to augment chloroplast ATP levels for maximal CO_2_ fixation. Instead, they reveal disequilibrium between thylakoid pmf and stromal MgATP^2−^ levels in vivo, with just 50% of the WT pmf sufficient to sustain ~100% of the maximum MgATP^2−^ level observed in our experimental setup, consistent with past in vitro work on intact chloroplasts^30,37^. An important caveat is that the TKTP-ATeam biosensor reports MgATP^2−^ rather than total adenylate charge, raising the possibility that stromal adenylate kinase (AK) in *pgr5CAS* buffers MgATP^2−^ via the reaction ADP + ADP ⇌ ATP + AMP^38^. However, chloroplast AK protein levels are unchanged in *pgr5*^12^, and significant AK activity would tend to restrict gH^+^ in *pgr5CAS* as ADP concentrations decreased, the opposite of what we and others observe^9,14^. Our data are therefore consistent with previous evidence that MgATP^2−^ and adenylate charge in chloroplasts are closely correlated^30,39,40^.

**Extended Data Fig. 3:**
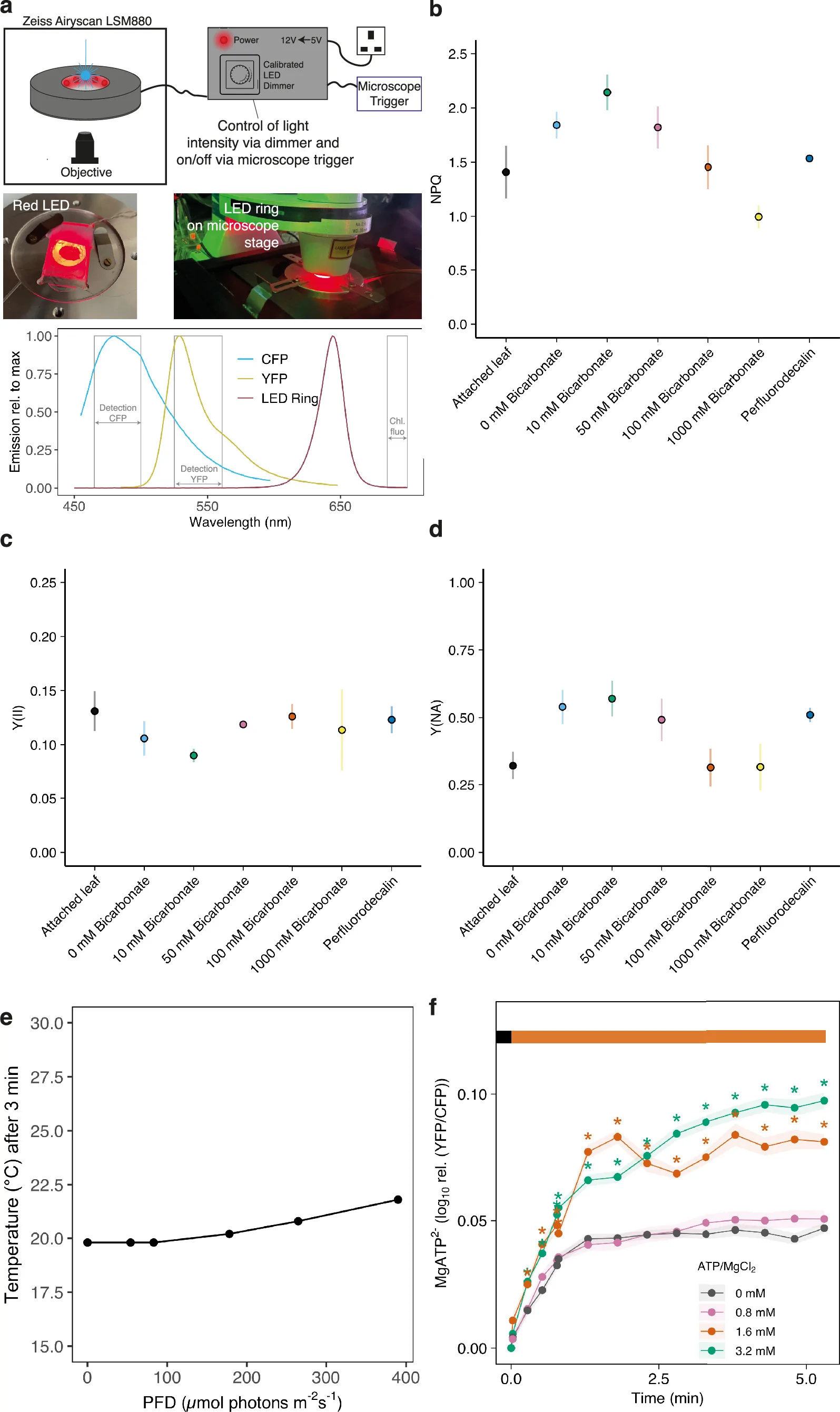
Supplementary Fig. 3: Experimental setup of the ATP biosensor. **a**) Top: Combination of the Zeiss Airyscan LSM880 with a red dimmable LED ring. The 12 V LEDs integrate with the Zeiss ZenBlack software via the Trigger.Out interface. This allows switching the LEDs on/off at desired times. Light intensities can be manually controlled via the dimmer up to a maximum light intensity of 390 µmol photons m^−2^ s^−1^. Bottom: Emission spectrum of the biosensor and LED ring and detection windows. **b-d**) Validation of the experimental setup to include 100 mM NaHCO_3_ to avoid CO_2_-limitation of the leaf mounted between two cover slips. At 100 mM NaHCO_3_, similar (**b**) NPQ, (**c**) Y(II) and (**d**) Y(NA) values of WT TKTP-ATeam to an attached leaf were achieved. **e**) Temperature effect of the LED on the leaf mounted between two cover slips. The temperature of the leaf was determined after 3 min at each light intensity, corresponding to the setup used in Fig. 2. **f**) Response of the ATP biosensor in WT-TA leaves infiltrated with equimolar concentrations of ATP and MgCl_2_ in the presence of 100 µM digitonin to partially permeabilise the cell membranes and to test the responsiveness of the sensor to ATP increase. Asterisks indicate significant differences between 0 mM and other ATP/MgCl_2_ treatments calculated from a one-way anova followed by a Tukey HSD test, confidence level = 0.95, (n = 24, 35, 13, 36).

How is the disequilibrium between thylakoid pmf and stromal MgATP^2−^ maintained? Our biosensor data suggest that ATP synthase activity is downregulated in the WT above a threshold MgATP^2−^ concentration, consistent with reports of so-called ‘metabolic control’ of gH^+^^41,42^, a regulatory mechanism that preserves ΔpH for PCON, NPQ and stromal alkalinization. The oligomycin infiltrations confirm this regulation can be overridden (Extended Data Fig. 5): inhibition of mitochondrial ATP synthase elevates cytosolic and hence chloroplastic Pi^36^, and our data are consistent with Pi itself acting as the metabolic regulator of chloroplast ATP synthase activity^9,43^. These experiments also confirm that the MgATP^2−^ levels in non-infiltrated leaves do not represent a ceiling in ATeam1.03-nD/nA biosensor sensitivity, as substantial additional dynamic range is clearly available (Extended Data Fig. 3f).

We next consider whether CBB cycle activity is itself attenuated in *pgr5/pgr5CAS*, which would reduce ATP demand and potentially mask a putative ATP production shortfall. We address this directly using an in-system readout of ATP consumption: the rate of stromal MgATP^2−^ decline in the dark immediately following illumination, which reflects continued CBB-driven ATP turnover once photophosphorylation has ceased. This post-illumination decay was similar in WT-TA and *pgr5CAS*-TA1 at matched light intensities under our experimental regime, which included 100 mM NaHCO_3_ (Fig. 1e,f), indicating that CBB-driven ATP demand is not appreciably decreased in *pgr5CAS*. The equivalent MgATP^2−^ levels in *pgr5CAS* therefore cannot be attributed to a compensatory fall in CBB ATP demand. This leaves open the basis of the lower CO_2_ fixation rate reported for *pgr5/pgr5CAS* under ambient CO_2_ and higher or fluctuating light^11–13^. Redox activation of CBB enzymes is WT-like in *pgr5*^44^, ruling out a thiol-mediated mechanism. However, the lower ΔpH characteristic of *pgr5/pgr5CAS* would necessarily attenuate the light-driven stromal alkalinization that brings stromal pH from ~7.2 in the dark to ~7.8 in the light^31,32^, potentially displacing stromal pH further from the alkaline optimum of rubisco and other CBB enzymes such as fructose-1,6-bisphosphatase and sedoheptulose-1,7-bisphosphatase^45,46^. We consider it likely that such ΔpH-dependent attenuation contributes to the decreased CO_2_ fixation observed under these more limiting conditions. A direct prediction of this model is that the post-illumination MgATP^2−^ decay should correspondingly slow in *pgr5CAS* at ambient CO_2_, where stromal alkalinization is rate-limiting for CBB flux, a prediction we identify for future testing. Either way, it is clear from our data that CO_2_ fixation in *pgr5CAS* is not limited by ATP production.

**Extended Data Fig. 4:**
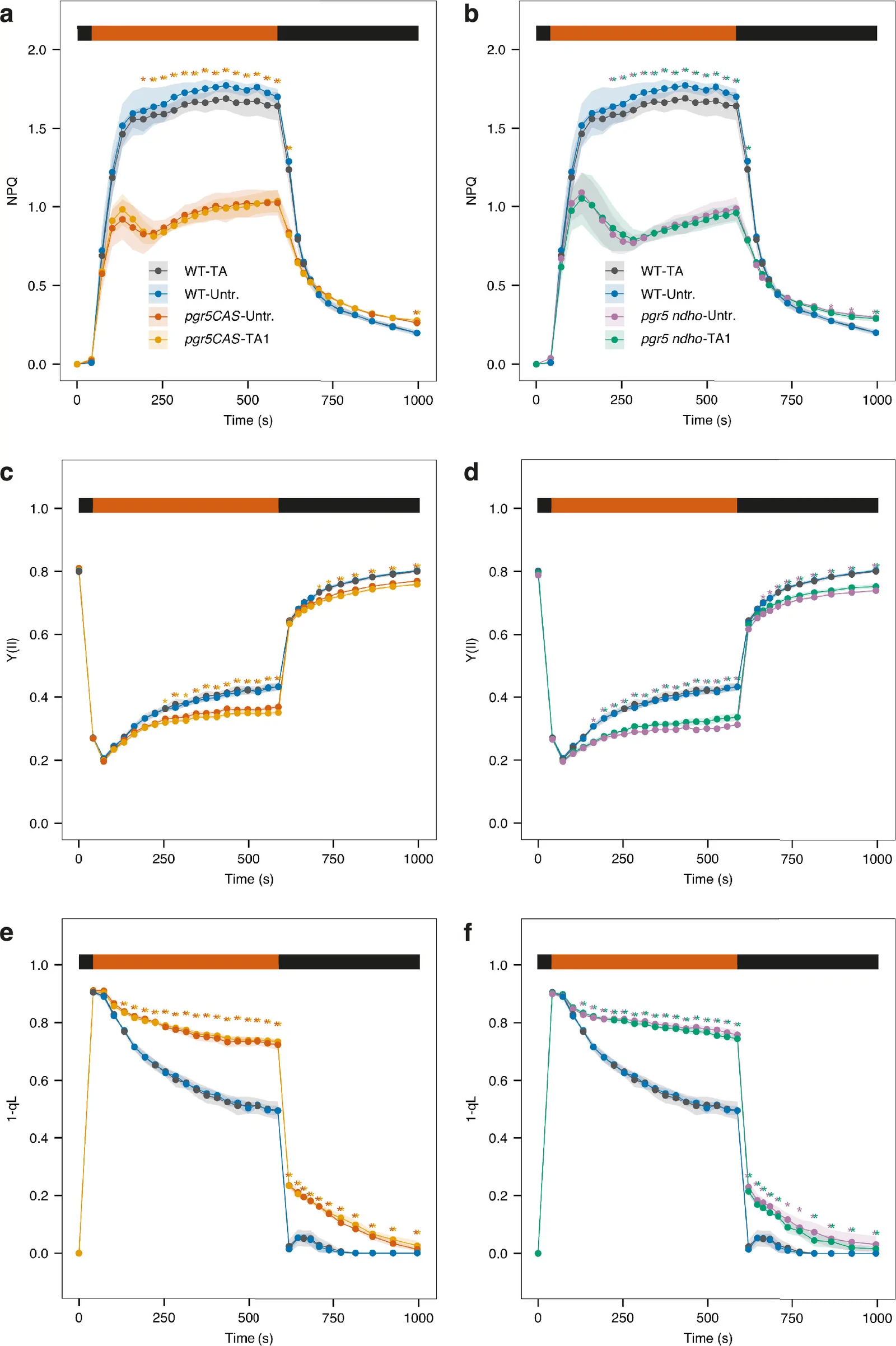
Supplementary Fig. 4: Photosynthetic parameters of untransformed (Untr.) and transformed (TA) WT, *pgr5CAS* and *pgr5 ndho* lines. (**a-b**) Yield of photosystem II (Y(II)). (**c-d**) PSII acceptor-side limitation (1-qL). (**e-f**) NPQ. For all panels, data points represent the mean of 3 biological replicates and shaded areas represent SEM. Asterisks indicate significant differences between WT-TA and other genotypes, calculated from a one-way anova followed by a Tukey HSD test, confidence level = 0.95. Coloured bar at the top of the panel indicates when red actinic light (401 µmol photons m^−2^ s^−1^) was turned on/off.

**Extended Data Fig. 5:**
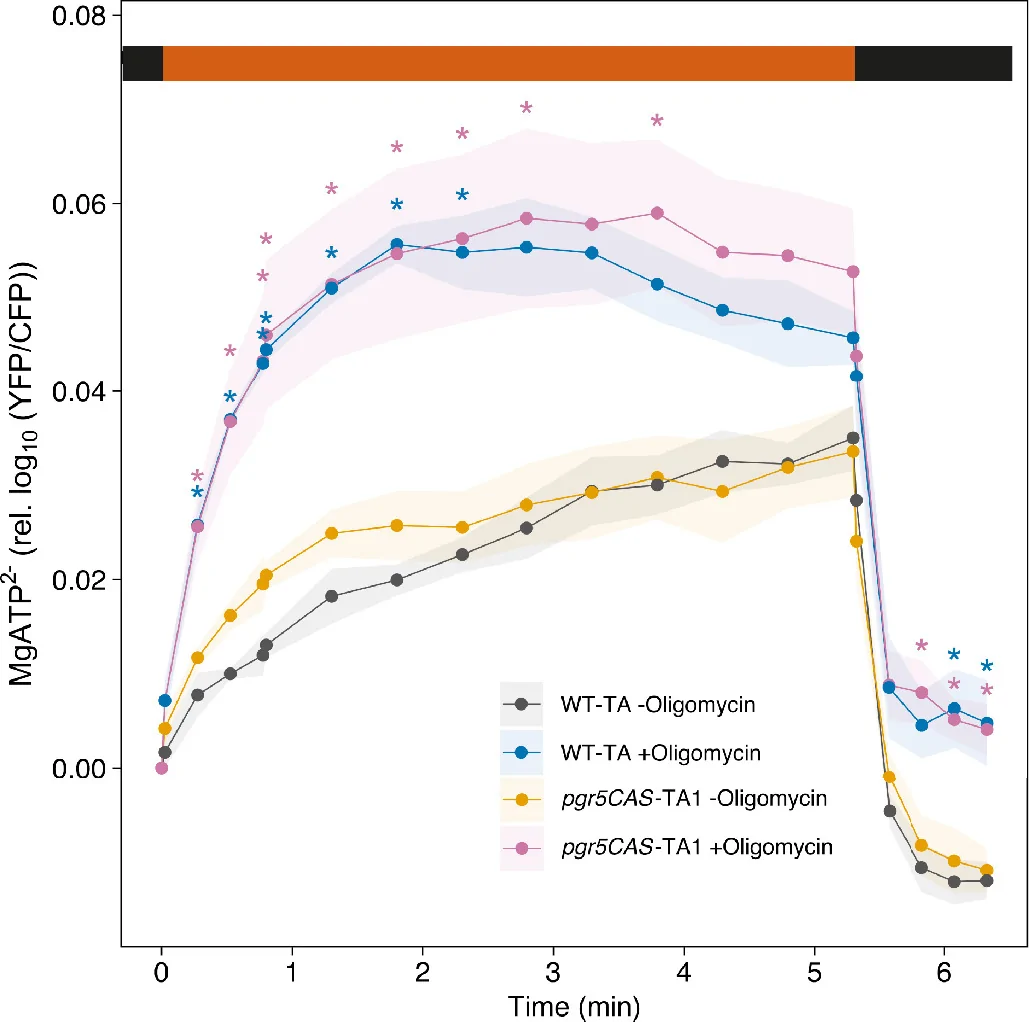
Supplementary Fig. 5: MgATP^2−^ dynamics in oligomycin-treated leaves after 5 min of illumination. Leaf infiltration buffer supplemented with or without 15 µM oligomycin was injected into leaves and returned to the growth chamber for 3 h. Leaves were then dark adapted for 1 h and measurements carried out as described previously. For all panels, data points represent the mean biological replicates (n = 3, 3, 4, 5) and shaded areas represent SEM. Asterisks indicate significant differences between WT-TA -Oligomycin and other genotypes, calculated from a one-way anova followed by a Tukey HSD test, confidence level = 0.95.

Our findings reframe our understanding of the *pgr5/pgr5CAS* and *pgr5 ndho* phenotypes: the photosensitivity under fluctuating light^20^, the impairment to growth under low or ambient CO_2_ conditions^11^ and the photosynthetic oscillations^13^ derive not from an ATP shortfall but from the loss of the additional ΔpH, which is required both to activate photoprotective processes (Extended Data Fig. 4) and to drive stromal alkalinization towards the optimum for CBB cycle activity^32,46^. The latter mechanism also revises our earlier interpretation of the *pgr5* oscillations^13^: rather than reflecting transient ATP shortfall, they more plausibly arise from disrupted ΔpH-dependent regulation of stromal pH, with the slow kinetics of stromal alkalinization during light transitions transiently slowing the CBB cycle and providing the feedback delay characteristic of oscillatory systems. Consistent with a ΔpH-centred model, the *pgr5* phenotype is ameliorated by processes that boost ΔpH such as flavodiiron-protein dependent pseudocyclic electron transfer^47^.

Our findings also bear on the broader question of how the chloroplast balances the ATP/NADPH stoichiometric mismatch between LET (1.28) and the CBB cycle (1.5). An established alternative to CET-mediated ATP augmentation is the export of excess reducing equivalents from the chloroplast via the malate valve^48,49^. The close correspondence between Y(II) and net CO_2_ assimilation observed across many C3 species^50^ has historically been taken to indicate that essentially all LET-derived reductants are consumed by the CBB cycle. However, recent biosensor measurements in *Arabidopsis* provide direct in vivo evidence that this reductant export from the chloroplast operates rapidly and at quantitatively significant rates during photosynthesis: the chloroplast-targeted TKTP-iNap NADPH biosensor reveals pronounced light-induced reduction of the stromal NADPH pool, while a cytosolic NADH/NAD^+^ biosensor shows a fast and pronounced reduction of the cytosolic NAD^+^ pool upon illumination, consistent with significant malate valve activity^26^. A stoichiometric shortfall of 0.22 ATP per NADPH would mandate that approximately 15% of LET-derived NADPH must be disposed of by export under purely carboxylating conditions to balance the stromal adenylate and pyridine nucleotide pools, a fraction well within the residual variance of the Y(II)–CO_2_ assimilation relationship in past measurements^50^. Under photorespiratory conditions, the chloroplast ATP/NADPH demand rises substantially above 1.5 (ref. ^51^), further increasing the fraction of stromal NADPH requiring export. Together, these considerations support a model in which the LET–CBB ATP/NADPH imbalance, exacerbated by photorespiration, is balanced primarily by exporting reducing equivalents via the malate valve rather than by augmenting chloroplast ATP via CET. These ideas now require further testing.

Our data do not resolve whether PGR5 acts primarily in CET or in direct regulation of ATP synthase activity, but they do constrain the mechanism: elevated gH^+^ in *pgr5CAS*-TA1 and *pgr5CAS*-TA2 is not accompanied by elevated MgATP^2−^ (Fig. 2h), excluding a productive proton leak. The *hope2* mutant, which also displays high gH^+^, compensates by upregulating PGR5-dependent CET and thereby maintains WT-like pmf and NPQ^12,13^, arguing for a role of PGR5 in proton influx rather than ATP synthase regulation. The elevated gH^+^ in *pgr5* itself may instead reflect altered ECS decay kinetics arising from Q-cycle mis-regulation at cytochrome *b*_6_*f* in the absence of PGR5^14,52^.

**Extended Data Fig. 6:**
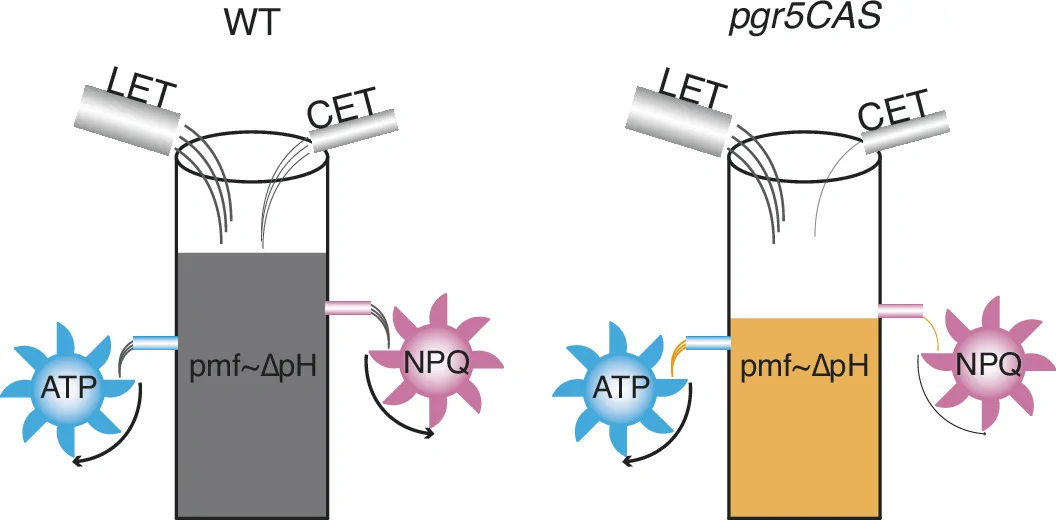
Supplementary Fig. 6: New role of PGR5-dependent CET. In the WT, LET and CET drive the build-up of sufficient pmf for synthesis of ATP and induction of NPQ. In *pgr5CAS*, lack of CET results in a lower pmf, sufficient for ATP synthesis but not induction of NPQ, resulting in photoinhibition.

In conclusion, we show that the primary role of CET is not in ATP augmentation for CO_2_ fixation. Rather, we propose that it provides the additional ΔpH required both for activating ΔpH-dependent photoprotective processes (PCON and NPQ) (summarized in Extended Data Fig. 6) and for driving stromal alkalinization to support optimal CBB cycle activity.

## Methods

### Generation of *pgr5CAS and pgr5CAS ndho* lines expressing TKTP-ATeam and plant growth

Seeds lacking the PGR5 protein^22^, (generated via CRISPR-Cas9 and referred to as *pgr5CAS*) and the *pgr5 ndho* double mutant (lacking PGR5 and the NDH complex^13^) were sterilized in 70% (v/v) EtOH and 0.1% (v/v) Triton-X 100 and stratified at 4°C for 48 h. Seeds were sowed on Levington Advance Seed and Modular F2S Compost + Sand and placed under long-day conditions (16 h light/8 h dark, 23/18 °C) and grown until flowering. For floral dip, *Agrobacterium tumefaciens* GV3101 cells were transformed with pEarleyGate100 containing the ATeam1.03-nD/nA sequence fused to the leader sequence from *Nicotiana tabacum* transketolase for targeting the plastid stroma under the control of a 35S promoter. T1 seeds were selected via resistance to glufosinate ammonium (120 mg l^−1^, 0.05% Silwet-77 (v/v)). Resistant plants with high TKTP-ATeam fluorescence were selected and backgrounds from two independent transformation events were taken to homozygosity and used for the analyses. The line *pgr5CAS-*TA1 exhibited the strongest fluorescence and displayed a low level of generational silencing. The second line *pgr5CAS*-TA2 was used for additional validation experiments (Extended Data Fig. 1). For *pgr5 ndho* two independent lines (*pgr5 ndho*-TA1 and *pgr5 ndho*-TA2) were selected for microscopy. For confocal imaging and photosynthetic measurements, *Arabidopsis thaliana* WT, *pgr5CAS* and *pgr5 ndho* expressing the TKTP-ATeam biosensor were grown in Levington Advance Seed and Modular F2S Compost + Sand under short-day conditions (9 h light/15 h dark, 23 °C/15 °C) for 4–5 weeks. Detached leaves were placed between two cover slips in the presence of 100 mM NaHCO_3_ as a CO_2_ source during confocal imaging and photosynthesis measurements (validation shown in Supplementary Fig. 3b–d).

### Confocal imaging

All confocal imaging was performed with a Zeiss Airyscan LSM880 using ZEN 2.3 (blue edition) in combination with a custom-built LED illumination chamber (Supplementary Fig. 3a). CFP was excited at 458 nm and emission was collected at 465–500 nm (CFP), 525–561 nm (YFP) and 670–690 nm for chlorophyll fluorescence. The pinhole was adjusted to 150 µm, pixel time to 2.05 μs, time to scan a full frame to 1.5 s and the resolution of the images was 512 × 512 pixels. The main beam splitter selected was the 458-Plate and a dichroic beamsplitter 1 was the mirror. For FRET measurements, an EC Plan-Neofluar 10×/0.3 objective was used, and for localization of the TKTP-ATeam biosensor to the chloroplast, a Plan-Apochromat 63×/1.4 Oil DIC M27 objective was used.

For time-series experiments, the ‘time series and experiment designer’ features were used, enabling the acquisition of images at specific time intervals. Turning the actinic light on/off during the time series was controlled via the software, whereas light intensity during a light curve was controlled manually via the dimmer switch. The dimmer intensity was calibrated before measurements to light intensities matching the WALZ DUAL PAM and DUAL KLAS machines.

Detached leaves were placed between two cover slips in the presence of 100 mM NaHCO_3_ to avoid CO_2_ limitation. This was placed in a custom-made LED illumination chamber, which was integrated with the trigger interface of the Zeiss Zen software as described previously^26^ (Supplementary Fig. 3a). Confocal images were processed with a MATLAB-based redox ratio analysis software^53^. First, the background signal was removed using the integrated functionality of the redox ratio analysis software. Then, for each biological replicate, 12 regions of interest (corresponding to 12 individual chloroplasts) were selected and the intensities of the single YFP and CFP channels were exported and used for further analysis and plotting in RStudio. The YFP:CFP intensity ratio was normalized to the value at time 0, the last time-point in the dark before onset of illumination and log_10_-transformed to approach the normal distribution. Spectra of the LEDs of the custom-built LED rings and Walz machines (described below) were determined using a LI-180 (LI-COR). To measure the impact of LED illumination on the temperature of leaves mounted between two cover slips, a thermocouple was placed between the cover slips onto the leaves and temperatures were measured at increasing light intensities after 3 min at each light intensity (Supplementary Fig. 3e).

### Photosynthesis measurements

The ECS at 515 nm was measured using a Dual-PAM analyser with a P515/535 emitter/detector module (Heinz Walz GmbH)^54^. Plants were dark adapted for at least 1 h before measurements, and leaves placed between two cover slips, as described above. The pmf was calculated from the amplitude of a single exponential decay fit to the first 500 ms of the ECS decay in the dark (ECS_t_), normalized to the height of a 50 μs single turnover flash (ECS_ST_) applied before the onset of actinic light. Conductance of the thylakoid membrane to protons (gH^+^) was calculated as the inverse of the time constant *τ*_ECS_ of the single exponential decay and proton flux was calculated as vH^+^ = pmf × gH^+^.

A Dual-KLAS-NIR photosynthesis analyser (Heinz Walz GmbH) was used for pulse-amplitude modulation chlorophyll fluorescence measurements and P700 absorption spectroscopy in the near-infrared^55^. Measurements were performed as described in ref. ^12^. In brief, after plants had dark adapted for at least 1 h, detached leaves were placed in 100 mM NaHCO_3_ between two cover slips and four pairs of pulse-modulated near-infrared measuring beams were zeroed and calibrated before each measurement. For each genotype, one leaf was used to generate differential model plots according to manufacturer’s protocol, which were used for online deconvolution to determine redox changes of P700. Before each measurement, maximum oxidation of P700 was determined by using the preprogrammed NIRmax routine. DCMU treatments for photosynthesis measurements were performed as described above.

### Inhibitor treatments

For DCMU measurements, detached leaves were incubated in the dark for 40 min in water containing 4 or 20 µM DCMU and 0.2% EtOH (v/v) or 0.2 % EtOH (v/v) as a mock control. For oligomycin treatments, leaf infiltration buffer (20 mM HEPES pH 7.5, 150 mM sorbitol and 50 mM NaCl) was supplemented with 15 µM oligomycin and injected into leaves. Plants were then returned to the growth chamber for 3 h and dark adapted for at least 1 h.

### Statistical analysis

Overall, 3–5 biological replicates were used for all experiments. Statistical analysis was performed in RStudio 2025.09.1+401 (R version 4.5.1). The aov function was used to perform a one-way ANOVA in conjunction with the Tukey HSD function to calculate the Tukey honest significant differences (HSD) for performing multiple pairwise-comparisons between the means of groups, with the confidence level set to 0.95. Asterisks indicate significant differences between either treatments or between WT-TA and other genotypes (indicated in the figure legends). Letters indicate significant differences between treatments or genotypes where indicated. Statistics for all figures is provided in Source Data Table 1.

### Reporting summary

Further information on research design is available in the Nature Portfolio Reporting Summary linked to this article.

## Supplementary information

Supplementary Information Supplementary Figs. 1–6.

Reporting Summary

Peer Review File

## Source data

Source Data Table 1 Statistical source data.

## Data Availability

The datasets used to generate Figs. 1–3 are available in Source Data Table 1. Additional data will be made available upon reasonable request. Source data are provided with this paper.

## References

[1] Allen, J. F. Photosynthesis of ATP—electrons, proton pumps, rotors, and poise. *Cell* **110**, 273–276 (2002). 12176312 10.1016/s0092-8674(02)00870-x

[2] Sacksteder, C. A., Kanazawa, A., Jacoby, M. E. & Kramer, D. M. The proton to electron stoichiometry of steady-state photosynthesis in living plants: a proton-pumping Q cycle is continuously engaged. *Proc. Natl Acad. Sci. USA* **97**, 14283–14288 (2000). 11121034 10.1073/pnas.97.26.14283 PMC18910

[3] Kramer, D. M. & Evans, J. R. The importance of energy balance in improving photosynthetic productivity. *Plant Physiol.* **155**, 70–78 (2010). 21078862 10.1104/pp.110.166652 PMC3075755

[4] Yamori, W. & Shikanai, T. Physiological functions of cyclic electron transport around photosystem I in sustaining photosynthesis and plant growth. *Annu. Rev. Plant Biol.* **67**, 1–26 (2015). 10.1146/annurev-arplant-043015-112002 26927905

[5] Munekage, Y. et al. Cyclic electron flow around photosystem I is essential for photosynthesis. *Nature* **429**, 579–582 (2004). 15175756 10.1038/nature02598

[6] Kobayashi, R., Yamamoto, H., Ishibashi, K. & Shikanai, T. Critical role of cyclic electron transport around photosystem I in the maintenance of photosystem I activity. *Plant J.* **118**, 2141–2153 (2024). 38558422 10.1111/tpj.16735

[7] Nakano, H., Yamamoto, H. & Shikanai, T. Contribution of NDH-dependent cyclic electron transport around photosystem I to the generation of proton motive force in the weak mutant allele of *pgr5*. *Biochim. Biophys. Acta - Bioenerg.* **1860**, 369–374 (2019). 30878346 10.1016/j.bbabio.2019.03.003

[8] Yamori, W., Shikanai, T. & Makino, A. Photosystem I cyclic electron flow via chloroplast NADH dehydrogenase-like complex performs a physiological role for photosynthesis at low light. *Sci Rep.* **5**, 13908 (2015). 26358849 10.1038/srep13908 PMC4566099

[9] Avenson, T. J., Cruz, J. A., Kanazawa, A. & Kramer, D. M. Regulating the proton budget of higher plant photosynthesis. *Proc. Natl Acad. Sci. USA* **102**, 9709–9713 (2005). 15972806 10.1073/pnas.0503952102 PMC1172270

[10] Wang, C., Yamamoto, H. & Shikanai, T. Role of cyclic electron transport around photosystem I in regulating proton motive force. *Biochim. Biophys. Acta - Bioenerg.* **1847**, 931–938 (2015). 10.1016/j.bbabio.2014.11.013 25481109

[11] Munekage, Y. N., Genty, B. & Peltier, G. Effect of PGR5 impairment on photosynthesis and growth in *Arabidopsis thaliana*. *Plant Cell Physiol.* **49**, 1688–1698 (2008). 18799484 10.1093/pcp/pcn140

[12] Degen, G. E. et al. High cyclic electron transfer via the PGR5 pathway in the absence of photosynthetic control. *Plant Physiol.* **192**, 370–386 (2023). 36774530 10.1093/plphys/kiad084 PMC10152662

[13] Degen, G. E., Pastorelli, F. & Johnson, M. P. Proton gradient regulation 5 is required to avoid photosynthetic oscillations during light transitions. *J. Exp. Bot.* **75**, 947–961 (2023). 10.1093/jxb/erad428 37891008

[14] Yamamoto, H. & Shikanai, T. Does the *Arabidopsis* proton gradient regulation 5 mutant leak protons from the thylakoid membrane? *Plant Physiol.* **184**, 421–427 (2020). 32636340 10.1104/pp.20.00850 PMC7479887

[15] Nikkanen, L. et al. PGR5 is needed for redox-dependent regulation of ATP synthase both in chloroplasts and in cyanobacteria. Preprint at *bioRxiv* 10.1101/2024.11.03.621747 (2025).

[16] Wilson, S., Johnson, M. P. & Ruban, A. V. Proton motive force in plant photosynthesis dominated by ΔpH in both low and high light. *Plant Physiol.* **187**, 263–275 (2021). 34618143 10.1093/plphys/kiab270 PMC8418402

[17] Degen, G. E. & Johnson, M. P. Photosynthetic control at the cytochrome *b*_6_*f* complex. *Plant Cell* **36**, 4065–4079 (2024). 38668079 10.1093/plcell/koae133 PMC11449013

[18] Murchie, E. H. & Ruban, A. V. Dynamic non-photochemical quenching in plants: from molecular mechanism to productivity. *Plant J.* **101**, 885–896 (2020). 31686424 10.1111/tpj.14601

[19] Munekage, Y. et al. PGR5 is involved in cyclic electron flow around photosystem I and is essential for photoprotection in *Arabidopsis*. *Cell* **110**, 361–371 (2002). 12176323 10.1016/s0092-8674(02)00867-x

[20] Suorsa, M. et al. Proton gradient regulation 5 is essential for proper acclimation of *Arabidopsis* photosystem i to naturally and artificially fluctuating light conditions. *Plant Cell* **24**, 2934–2948 (2012). 22822205 10.1105/tpc.112.097162 PMC3426124

[21] Penzler, J.-F. et al. A *pgr5* suppressor screen uncovers two distinct suppression mechanisms and links cytochrome *b*_6_*f* complex stability to PGR5. *Plant Cell* **36**, 4245–4266 (2024). 38781425 10.1093/plcell/koae098 PMC11449078

[22] Penzler, J.-F. et al. Commonalities and specialties in photosynthetic functions of proton gradient regulation 5 variants in *Arabidopsis*. *Plant Physiol.* **190**, 1866–1882 (2022). 35946785 10.1093/plphys/kiac362 PMC9614465

[23] De Col, V. et al. ATP sensing in living plant cells reveals tissue gradients and stress dynamics of energy physiology. *eLife* **6**, e26770 (2017). 28716182 10.7554/eLife.26770 PMC5515573

[24] Voon, C. P. et al. ATP compartmentation in plastids and cytosol of *Arabidopsis thaliana* revealed by fluorescent protein sensing. *Proc. Natl. Acad. Sci. USA* **115**, E10778–E10787 (2018). 30352850 10.1073/pnas.1711497115 PMC6233094

[25] Heineke, D. et al. Redox transfer across the inner chloroplast envelope membrane. *Plant Physiol.* **95**, 1131–1137 (1991). 16668101 10.1104/pp.95.4.1131 PMC1077662

[26] Zheng, K. et al. Advanced illumination-imaging reveals photosynthesis-triggered pH, ATP and NAD redox signatures across plant cell compartments. Preprint at *bioRxiv* 10.1101/2025.06.16.659786 (2025).

[27] Imamura, H. et al. Visualization of ATP levels inside single living cells with fluorescence resonance energy transfer-based genetically encoded indicators. *Proc. Natl Acad. Sci. USA* **106**, 15651–15656 (2009). 19720993 10.1073/pnas.0904764106 PMC2735558

[28] Heldt, H. W., Werdan, K., Milovancev, M. & Geller, G. Alkalization of the chloroplast stroma caused by light-dependent proton flux into the thylakoid space. *Biochim. Biophys. Acta - Bioenerg.* **314**, 224–241 (1973). 10.1016/0005-2728(73)90137-0 4747067

[29] Su, P.-H. & Lai, Y.-H. A reliable and non-destructive method for monitoring the stromal pH in isolated chloroplasts using a fluorescent pH probe. *Front. Plant Sci.* **8**, 2079 (2017). 29259618 10.3389/fpls.2017.02079 PMC5723387

[30] Giersch, C. et al. Energy charge, phosphorylation potential and proton motive force in chloroplasts. *Biochim. Biophys. Acta - Bioenerg.* **590**, 59–73 (1980). 10.1016/0005-2728(80)90146-2 7356995

[31] Werdan, K. & Heldt, H. W. Accumulation of bicarbonate in intact chloroplasts following a pH gradient. *Biochim. Biophys. Acta - Bioenerg.* **283**, 430–441 (1972). 10.1016/0005-2728(72)90260-5 4630890

[32] Werdan, K., Heldt, H. W. & Milovancev, M. The role of pH in the regulation of carbon fixation in the chloroplast stroma. Studies on CO_2_ fixation in the light and dark. *Biochim. Biophys. Acta - Bioenerg.* **396**, 276–292 (1975). 10.1016/0005-2728(75)90041-9 239746

[33] Robinson, S. P. The involvement of stromal ATP in maintaining the pH gradient across the chloroplast envelope in the light. *Biochim. Biophys. Acta - Bioenerg.* **806**, 187–194 (1985).

[34] Dang, K.-V. et al. Combined increases in mitochondrial cooperation and oxygen photoreduction compensate for deficiency in cyclic electron flow in *Chlamydomonas reinhardtii*. *Plant Cell* **26**, 3036–3050 (2014). 24989042 10.1105/tpc.114.126375 PMC4145130

[35] Alber, N. A. & Vanlerberghe, G. C. The flexibility of metabolic interactions between chloroplasts and mitochondria in *Nicotiana tabacum* leaf. *Plant J.* **106**, 1625–1646 (2021). 33811402 10.1111/tpj.15259

[36] Chadee, A., Alber, N. A., Dahal, K. & Vanlerberghe, G. C. The complementary roles of chloroplast cyclic electron transport and mitochondrial alternative oxidase to ensure photosynthetic performance. *Front. Plant Sci.* **12**, 748204 (2021). 34650584 10.3389/fpls.2021.748204 PMC8505746

[37] Backhausen, J. E., Kitzmann, C., Horton, P. & Scheibe, R. Electron acceptors in isolated intact spinach chloroplasts act hierarchically to prevent over-reduction and competition for electrons. *Photosynth. Res.* **64**, 1–13 (2000). 16228439 10.1023/A:1026523809147

[38] Lange, P. R., Geserick, C., Tischendorf, G. & Zrenner, R. Functions of chloroplastic adenylate kinases in *Arabidopsis*. *Plant Physiol.* **146**, 323–324 (2007). 10.1104/pp.107.114702 PMC2245825 18162585

[39] Heber, U., Takahama, U., Neimanis, S. & Shimizu-Takahama, M. Transport as the basis of the kok effect. Levels of some photosynthetic intermediates and activation of light-regulated enzymes during photosynthesis of chloroplasts and green leaf protoplasts. *Biochim. Biophys. Acta - Bioenerg.* **679**, 287–299 (1982).

[40] Santarius, K. A. & Heber, U. Changes in the intracellular levels of ATP, ADP, AMP and Pi and regulatory function of the adenylate system in leaf cells during photosynthesis. *Biochim. Biophys. Acta - Biophys. Incl. Photosynth.* **102**, 39–54 (1965). 10.1016/0926-6585(65)90201-3 5833412

[41] Kanazawa, A. et al. Chloroplast ATP synthase modulation of the thylakoid proton motive force: implications for photosystem I and photosystem II photoprotection. *Front. Plant Sci.* **8**, 719 (2017). 28515738 10.3389/fpls.2017.00719 PMC5413553

[42] Kanazawa, A. & Kramer, D. M. In vivo modulation of nonphotochemical exciton quenching (NPQ) by regulation of the chloroplast ATP synthase. *Proc. Natl Acad. Sci. USA* **99**, 12789–12794 (2002). 12192092 10.1073/pnas.182427499 PMC130538

[43] Takizawa, K., Kanazawa, A. & Kramer, D. M. Depletion of stromal P_i_ induces high ‘energy-dependent’ antenna exciton quenching (q_E_) by decreasing proton conductivity at CF_O_-CF_1_ ATP synthase. *Plant Cell Environ.* **31**, 235–243 (2008). 17996016 10.1111/j.1365-3040.2007.01753.x

[44] Okegawa, Y., Tsuda, N., Sakamoto, W. & Motohashi, K. Maintaining the chloroplast redox balance through the PGR5-dependent pathway and the Trx system is required for light-dependent activation of photosynthetic reactions. *Plant Cell Physiol.* **63**, 92–103 (2021). 10.1093/pcp/pcab148 34623443

[45] Mott, K. A. & Berry, J. A. Effects of pH on activity and activation of ribulose 1,5-bisphosphate carboxylase at air level CO_2_. *Plant Physiol.* **82**, 77–82 (1986). 16665027 10.1104/pp.82.1.77 PMC1056069

[46] Lorimer, G. H., Badger, M. R. & Andrews, T. J. The activation of ribulose-1,5-bisphosphate carboxylase by carbon dioxide and magnesium ions. Equilibria, kinetics, a suggested mechanism, and physiological implications. *Biochemistry* **15**, 529–536 (1976). 3199 10.1021/bi00648a012

[47] Yamamoto, H., Takahashi, S., Badger, M. R. & Shikanai, T. Artificial remodelling of alternative electron flow by flavodiiron proteins in *Arabidopsis*. *Nat. Plants* **2**, 16012 (2016). 27249347 10.1038/nplants.2016.12

[48] Scheibe, R. Malate valves to balance cellular energy supply. *Physiol. Plant.* **120**, 21–26 (2004). 15032873 10.1111/j.0031-9317.2004.0222.x

[49] Selinski, J. & Scheibe, R. Malate valves: old shuttles with new perspectives. *Plant Biol.* **21**, 21–30 (2019). 29933514 10.1111/plb.12869 PMC6586076

[50] Genty, B., Briantais, J.-M. & Baker, N. R. The relationship between the quantum yield of photosynthetic electron transport and quenching of chlorophyll fluorescence. *Biochim. Biophys. Acta - Gen. Subj.* **990**, 87–92 (1989).

[51] Lim, S.-L. et al. In planta study of photosynthesis and photorespiration using NADPH and NADH/NAD^+^ fluorescent protein sensors. *Nat. Commun.* **11**, 3238 (2020). 32591540 10.1038/s41467-020-17056-0 PMC7320160

[52] Buchert, F., Mosebach, L., Gäbelein, P. & Hippler, M. PGR5 is required for efficient Q cycle in the cytochrome *b*_6_*f* complex during cyclic electron flow. *Biochem. J.* **477**, 1631–1650 (2020). 32267468 10.1042/BCJ20190914

[53] Fricker, M. D. Quantitative redox imaging software. *Antioxid. Redox Signal.* **24**, 752–762 (2016). 26154420 10.1089/ars.2015.6390

[54] Klughammer, C., Siebke, K. & Schreiber, U. Continuous ECS-indicated recording of the proton-motive charge flux in leaves. *Photosynth. Res.* **117**, 471–487 (2013). 23860827 10.1007/s11120-013-9884-4 PMC3825596

[55] Klughammer, C. & Schreiber, U. Deconvolution of ferredoxin, plastocyanin, and P700 transmittance changes in intact leaves with a new type of kinetic LED array spectrophotometer. *Photosynth. Res.* **128**, 195–214 (2016). 26837213 10.1007/s11120-016-0219-0 PMC4826414

