# Supplementary material for: Disequilibrium between chloroplast proton motive force and ATP levels in Arabidopsis
Source: Nat Plants. 2026 Aug 17;12(9):1679–87. doi: 10.1038/s41477-026-02374-w (PMC13585562; doi:10.1038/s41477-026-02374-w)
Supplement: Supplementary file 1 [file 41477_2026_2374_MOESM1_ESM.pdf]

nature plants

Supplementary information

https://doi.org/10.1038/s41477-026-02374-w

Disequilibrium between chloroplast proton motive force and ATP levels in *Arabidopsis*

In the format provided by the authors and unedited

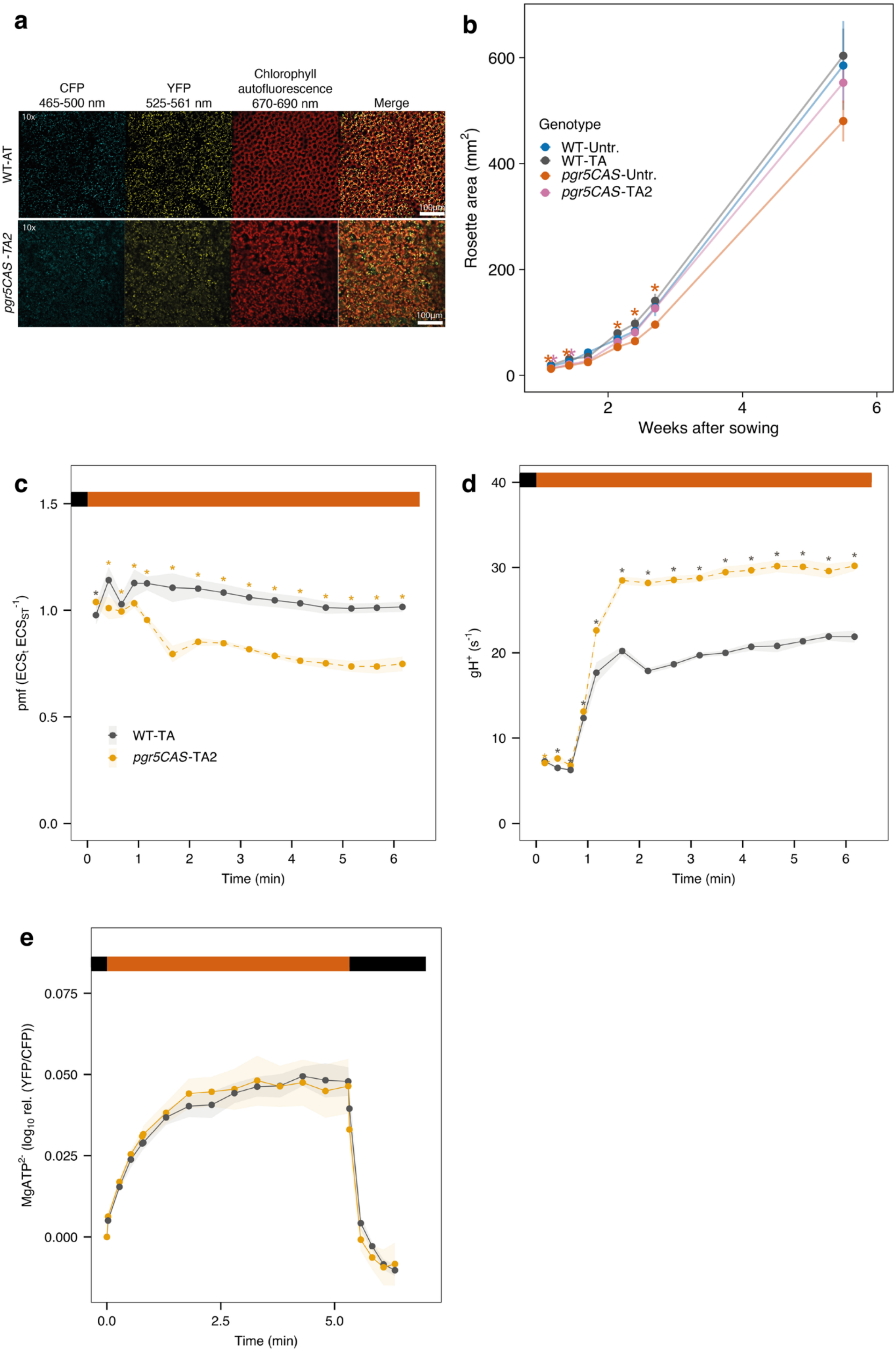

a
CFP 465-500 nm
YFP 525-561 nm
Chlorophyll autofluorescence 670-690 nm
Merge
WT-AT
pgr5CAS-TA2
10x
100μm
b
Genotype
WT-Untr.
WT-TA
pgr5CAS-Untr.
pgr5CAS-TA2
Rosette area (mm²)
Weeks after sowing
c
pmf (ECSt ECSST-1)
WT-TA
pgr5CAS-TA2
Time (min)
d
gH+ (s-1)
Time (min)
e
MgATP2- (log10 rel. (YFP/CFP))
Time (min)

Supplementary Figure 1: Validation of a second *pgr5CAS*-TA2. a) Confocal microscopy of WT-TA and *pgr5CAS*-TA2 showing the stable expression of the chloroplast stroma-localised FRET $MgATP^{2-}$ biosensors ATeam1.03-nD/nA. b) Rosette area between untransformed and transformed WT and *pgr5CAS* lines, (n = 6, 6, 6, 12). c) pmf in WT-TA and *pgr5CAS*-TA2, (n = 3, 5). d) gH^+ in WT-TA and *pgr5CAS*-TA2, (n = 3, 5). e) $MgATP^{2-}$ levels in WT-TA and *pgr5CAS*-TA2, (n = 4, 3). For all panels, data points represent the mean of biological replicates (n indicated above) and shaded areas/error bars represent SEM. Asterisks indicate significant differences between WT-TA and *pgr5CAS*-TA2, calculated from a one-way anova followed by a Tukey HSD test, confidence level = 0.95.

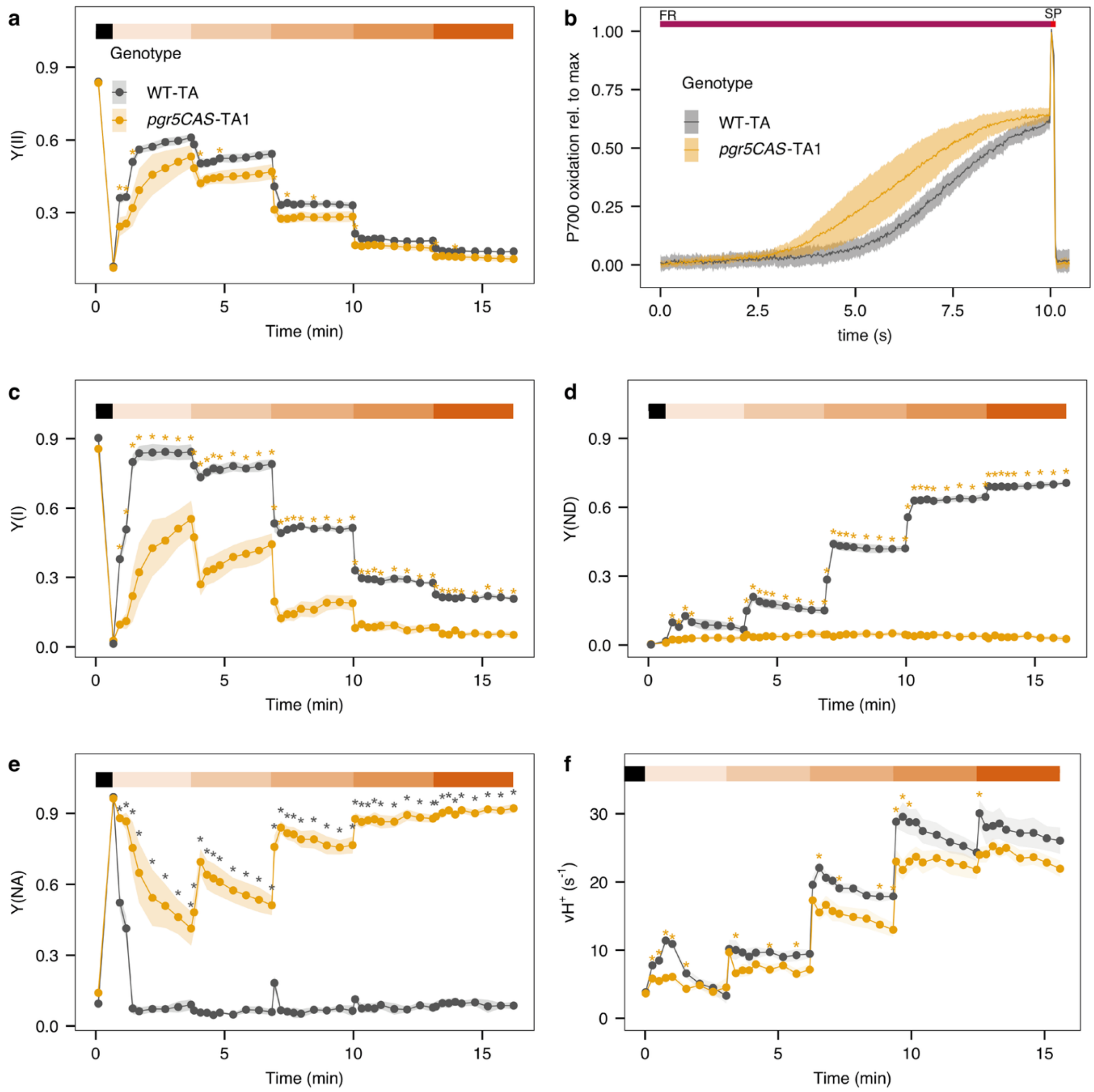

Supplementary Figure 2: Photosynthetic parameters of WT TKTP-ATeam and *pgr5CAS*-TA1 in response to increasing light intensities. a) Yield of photosystem II (Y(II)), (n = 4). b) Oxidation of P700 under far red light normalised to a saturating flash at 10s, (n = 4). c) Yield of photosystem I (Y(I)), (n = 4) d) Donor-side limitation of PSI, also referred to as P700 oxidation (Y(ND)), (n = 4) e) Acceptor-side limitation of PSI, also referred to as P700 reduction (Y(NA)), (n = 4). f) Proton flux through the thylakoid membrane, (n = 4, 7). For all panels, data points represent the mean of biological replicates (n indixated above) and shaded areas represent SEM. Asterisks indicate significant differences between WT-TA and other genotypes, calculated from a one-way anova followed by a Tukey HSD test, confidence level = 0.95. Coloured bars at the top of the panel indicate red actinic light intensities. The same light intensities as in Fig 2 a-d were used.

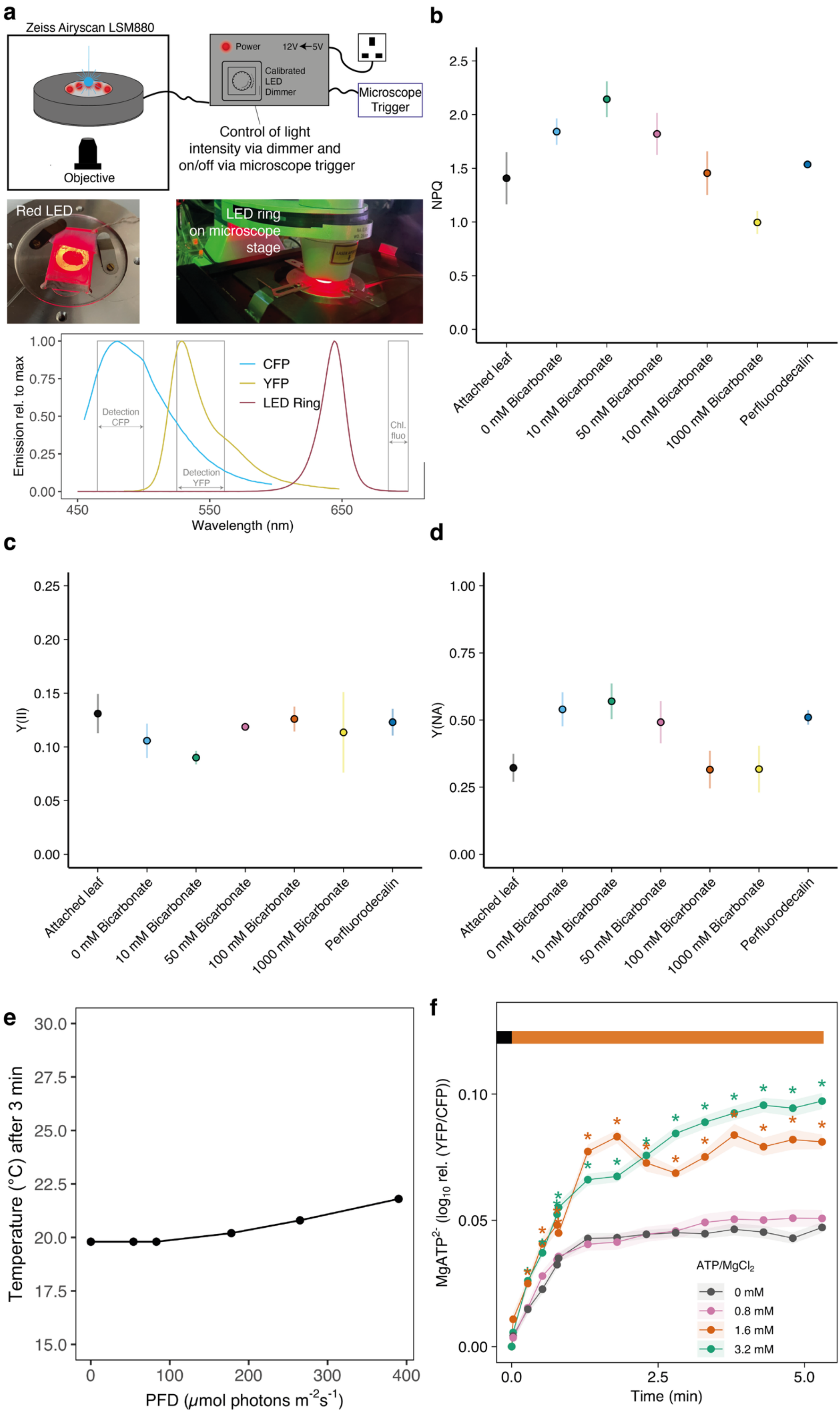

a
Zeiss Airyscan LSM880
Objective
Power
12V
5V
Calibrated
LED
Dimmer
Microscope
Trigger
Control of light
intensity via dimmer and
on/off via microscope trigger
Red LED
LED ring
on microscope
stage
Emission rel. to max
Wavelength (nm)
Detection
CFP
Detection
YFP
Chl.
fluo
CFP
YFP
LED Ring
b
NPQ
c
Y(II)
d
Y(NA)
Attached leaf
0 mM Bicarbonate
10 mM Bicarbonate
50 mM Bicarbonate
100 mM Bicarbonate
1000 mM Bicarbonate
Perfluorodecalin
e
Temperature (°C) after 3 min
PFD (μmol photons m-2s-1)
f
MgATP2- (log10 rel. (YFP/CFP))
Time (min)
ATP/MgCl2
0 mM
0.8 mM
1.6 mM
3.2 mM

Supplementary Figure 3: Experimental setup of the ATP biosensor. a) Top: Combination of the Zeiss Airyscan LSM880 with a red dimmable LED ring. The 12V LEDs integrate with the Zeiss ZenBlack software via the Trigger.Out interface. This allows switching the LEDs on/off at desired times. Light intensities can be manually controlled via the dimmer up to a maximum light intensity of 390 µmol photons m^{-2} s^{-1}. Bottom: Emission spectrum of the biosensor and LED ring and detection windows. b-d) Validation of the experimental setup to include 100 mM $NaHCO_3$ to avoid CO_2-limitation of the leaf mounted between two cover slips. At 100 mM $NaHCO_3$, similar (b) NPQ, (c) Y(II) and (d) Y(NA) values of WT TKTP-ATeam to an attached leaf were achieved. e) Temperature effect of the LED on the leaf mounted between two cover slips. The temperature of the leaf was determined after 3 min at each light intensity, corresponding to the setup used in Fig 2. f) Response of the ATP biosensor in WT-TA leaves infiltrated with equimolar concentrations of ATP and $MgCl_2$ in the presence of 100 µM digitonin to partially permeabilise the cell membranes and to test the responsiveness of the sensor to ATP increase. Asterisks indicate significant differences between 0mM and other ATP/$MgCl_2$ treatments calculated from a one-way anova followed by a Tukey HSD test, confidence level = 0.95, (n = 24, 35, 13, 36).

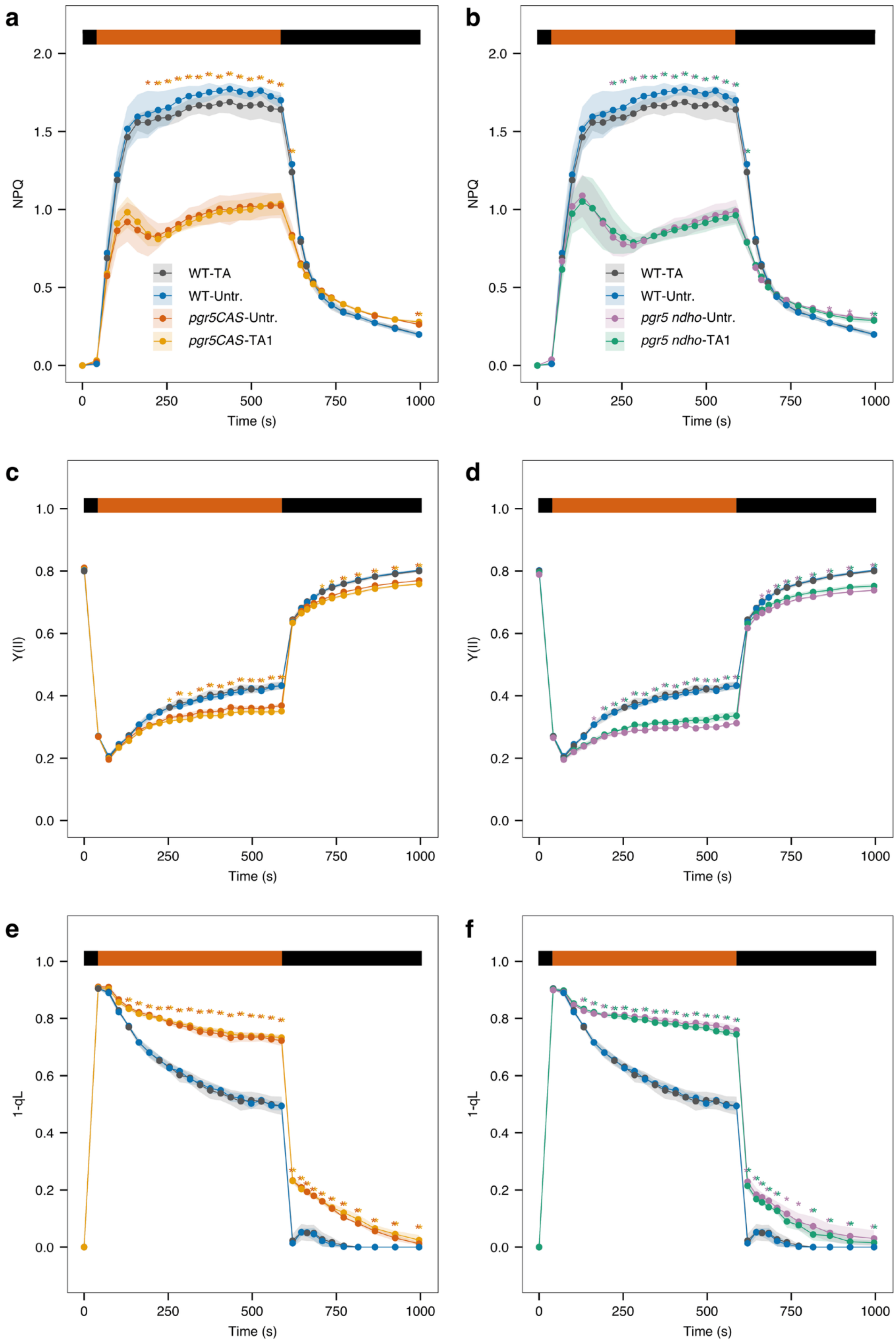
a
b
c
d
e
f
NPQ
Y(II)
1-qL
Time (s)
WT-TA
WT-Untr.
pgr5CAS-Untr.
pgr5CAS-TA1
pgr5 ndho-Untr.
pgr5 ndho-TA1

Supplementary Figure 4: Photosynthetic parameters of untransformed (Untr.) and transformed (TA) WT, *pgr5CAS* and *pgr5 ndho* lines. (a-b) Yield of photosystem Y(II). (c-d) PSII acceptor-side limitation (1-qL). (e-f) NPQ.
For all panels, data points represent the mean of 3 biological replicates and shaded areas represent SEM. Asterisks indicate significant differences between WT-TA and other genotypes, calculated from a one-way anova followed by a Tukey HSD test, confidence level = 0.95. Coloured bar at the top of the panel indicates when red actinic light (401 µmol photons m^{-2} s^{-1}) was turned on/off.

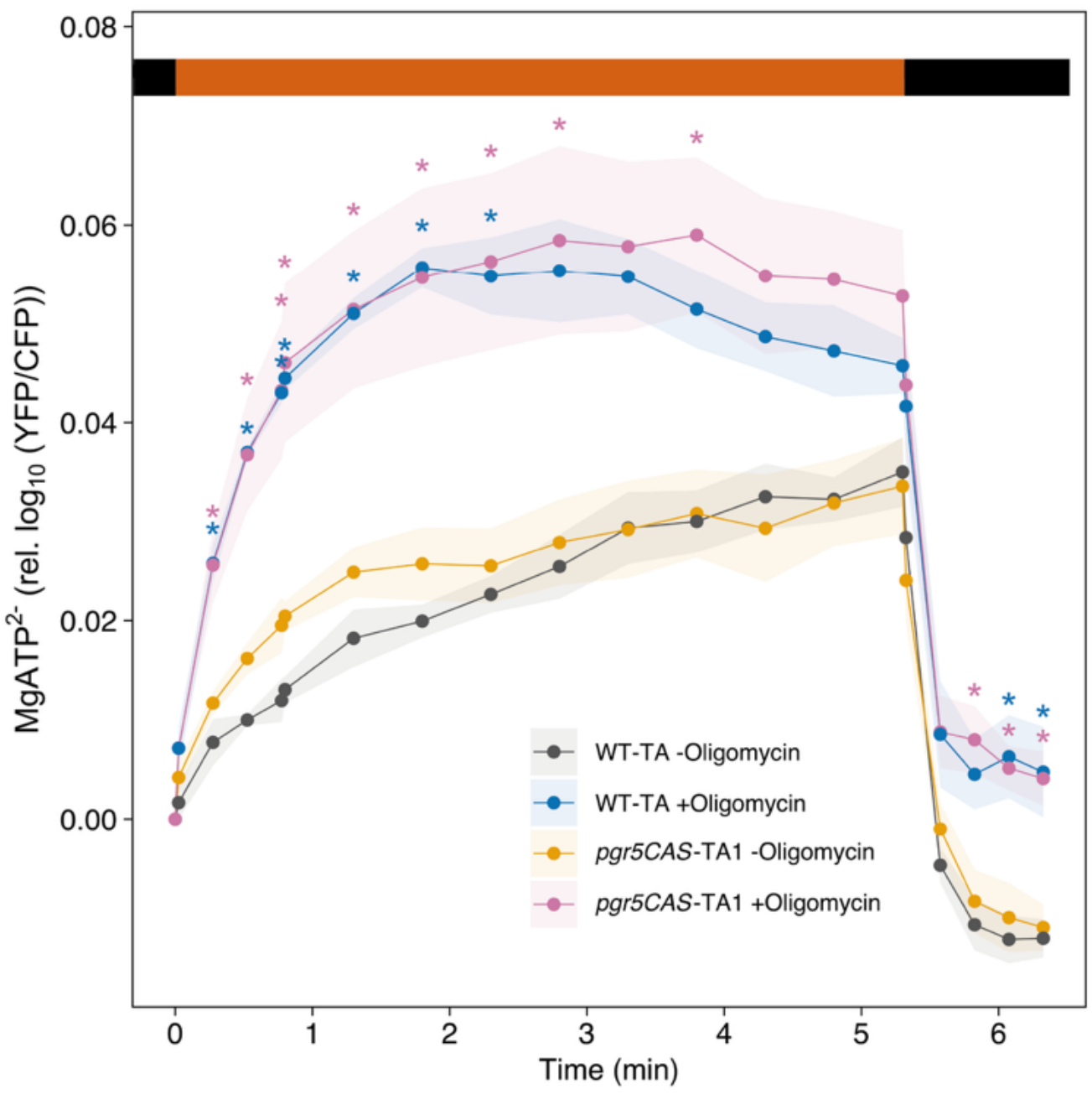

Supplementary Figure 5: $MgATP^{2-}$ dynamics in oligomycin-treated leaves after 5 min of illumination. Leaf infiltration buffer supplemented with or without 15 µM oligomycin was injected into leaves and returned to the growth chamber for 3 h. Leaves were then dark-adapted for 1h and measurements carried out as described previously. For all panels, data points represent the mean biological replicates (n = 3, 3, 4, 5) and shaded areas represent SEM. Asterisks indicate significant differences between WT-TA -Oligomycin and other genotypes, calculated from a one-way anova followed by a Tukey HSD test, confidence level = 0.95.

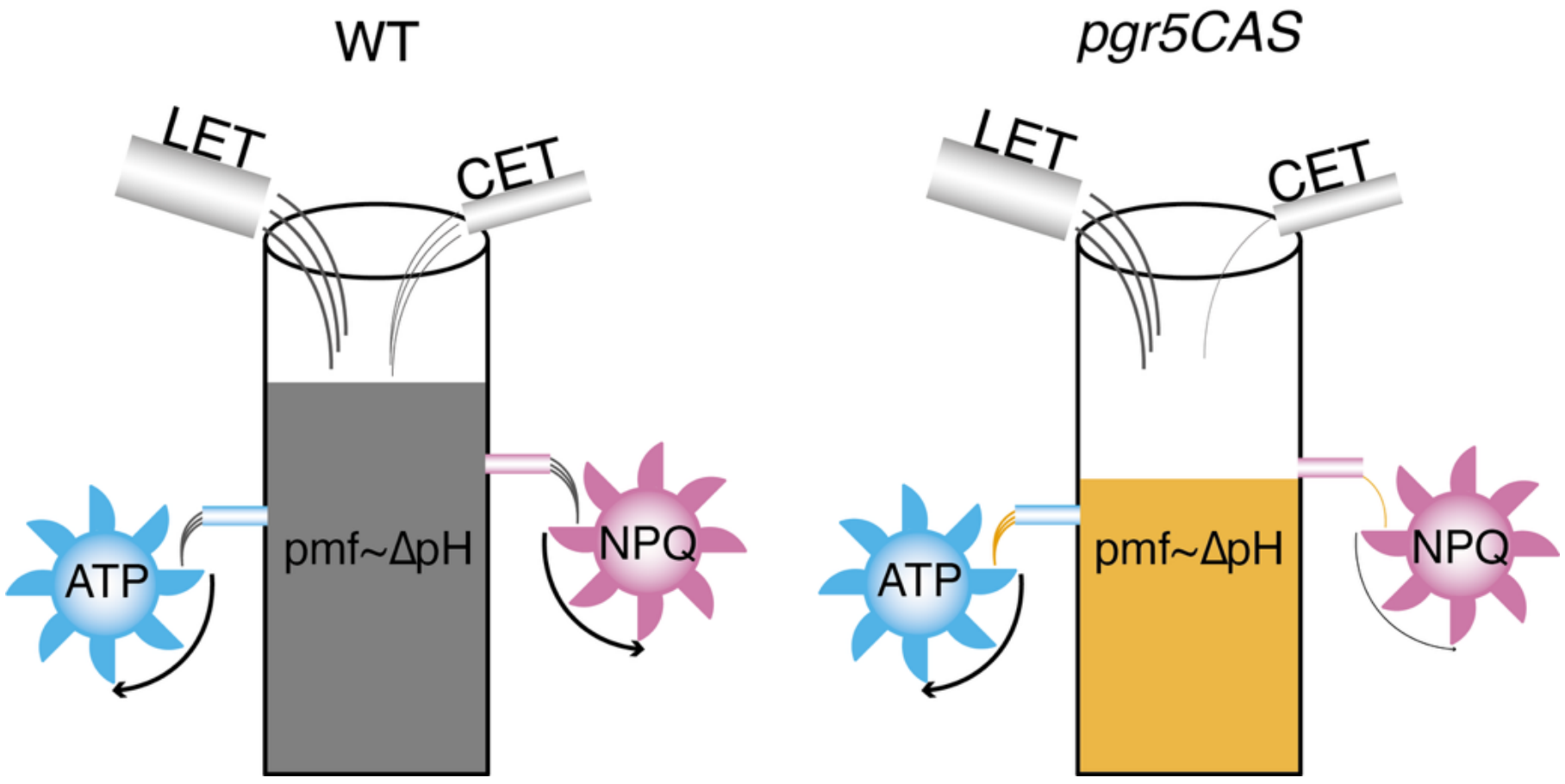

Supplementary Figure 6: New role of PGR5-dependent CET. In the WT, LET and CET drive the build-up of sufficient pmf for synthesis of ATP and induction of NPQ. In *pgr5CAS*, lack of CET results in a lower pmf, sufficient for ATP synthesis but not induction of NPQ, resulting in photoinhibition.
