# Supplementary material for: Disequilibrium between chloroplast proton motive force and ATP levels in Arabidopsis
Source: Nat Plants. 2026 Aug 17;12(9):1679–87. doi: 10.1038/s41477-026-02374-w (PMC13585562; doi:10.1038/s41477-026-02374-w)
Supplement: Supplementary file 2 [file 41477_2026_2374_MOESM2_ESM.pdf]

nature portfolio

Corresponding author(s): Gustaf E. Degen, Matthew P. Johnson

Last updated by author(s): 25.06.2026

Reporting Summary

Nature Portfolio wishes to improve the reproducibility of the work that we publish. This form provides structure for consistency and transparency in reporting. For further information on Nature Portfolio policies, see our Editorial Policies and the Editorial Policy Checklist.

Statistics

For all statistical analyses, confirm that the following items are present in the figure legend, table legend, main text, or Methods section.

n/a	Confirmed	
☐	☒	The exact sample size (*n*) for each experimental group/condition, given as a discrete number and unit of measurement
☒	☐	A statement on whether measurements were taken from distinct samples or whether the same sample was measured repeatedly
☐	☒	The statistical test(s) used AND whether they are one- or two-sided<br>*Only common tests should be described solely by name; describe more complex techniques in the Methods section.*
☒	☐	A description of all covariates tested
☒	☐	A description of any assumptions or corrections, such as tests of normality and adjustment for multiple comparisons
☐	☒	A full description of the statistical parameters including central tendency (e.g. means) or other basic estimates (e.g. regression coefficient) AND variation (e.g. standard deviation) or associated estimates of uncertainty (e.g. confidence intervals)
☐	☒	For null hypothesis testing, the test statistic (e.g. *F*, *t*, *r*) with confidence intervals, effect sizes, degrees of freedom and *P* value noted<br>*Give P values as exact values whenever suitable.*
☒	☐	For Bayesian analysis, information on the choice of priors and Markov chain Monte Carlo settings
☒	☐	For hierarchical and complex designs, identification of the appropriate level for tests and full reporting of outcomes
☒	☐	Estimates of effect sizes (e.g. Cohen's *d*, Pearson's *r*), indicating how they were calculated

Our web collection on statistics for biologists contains articles on many of the points above.

Software and code

Policy information about availability of computer code

Data collection	For microscopy: Zen Black 2.3 was used. For chlorophyll fluorescence Dual KLAS software version 3.016. For ECS Dual PAM software version 3.28.
Data analysis	For Rstudio version 2025.09.1+401 (R version 4.5.1). A custom code was written to fit the ECS data with an exponential decay function to calculate the pmf and gH+ parameters.

For manuscripts utilizing custom algorithms or software that are central to the research but not yet described in published literature, software must be made available to editors and reviewers. We strongly encourage code deposition in a community repository (e.g. GitHub). See the Nature Portfolio guidelines for submitting code & software for further information.

Data

Policy information about availability of data

All manuscripts must include a data availability statement. This statement should provide the following information, where applicable:

- Accession codes, unique identifiers, or web links for publicly available datasets
- A description of any restrictions on data availability
- For clinical datasets or third party data, please ensure that the statement adheres to our policy

Data used to generate the figures is showin in Extended Table 1. Other data will be provided upon reasonable request.

Research involving human participants, their data, or biological material

Policy information about studies with human participants or human data. See also policy information about sex, gender (identity/presentation), and sexual orientation and race, ethnicity and racism.

Reporting on sex and gender	NA
Reporting on race, ethnicity, or other socially relevant groupings	NA
Population characteristics	NA
Recruitment	NA
Ethics oversight	NA

Note that full information on the approval of the study protocol must also be provided in the manuscript.

Field-specific reporting

Please select the one below that is the best fit for your research. If you are not sure, read the appropriate sections before making your selection.

☒ Life sciences ☐ Behavioural & social sciences ☐ Ecological, evolutionary & environmental sciences

For a reference copy of the document with all sections, see nature.com/documents/nr-reporting-summary-flat.pdf

Life sciences study design

All studies must disclose on these points even when the disclosure is negative.

Sample size	A sample size of >3 biological replicates were used, common in the field of plant biology to generate statistically significant results.
Data exclusions	No data was excluded.
Replication	Measurements with the ATeam biosensor were carried out for two indepdendently transformed pgr5CAS and two pgr5 ndho lines, yielding similar results.
Randomization	The order of plants was randomised during data collection.
Blinding	NA

Reporting for specific materials, systems and methods

We require information from authors about some types of materials, experimental systems and methods used in many studies. Here, indicate whether each material, system or method listed is relevant to your study. If you are not sure if a list item applies to your research, read the appropriate section before selecting a response.

Materials & experimental systems

n/a	Involved in the study	
☒	☐	Antibodies
☒	☐	Eukaryotic cell lines
☒	☐	Palaeontology and archaeology
☒	☐	Animals and other organisms
☒	☐	Clinical data
☒	☐	Dual use research of concern
☐	☒	Plants

Methods

n/a	Involved in the study	
☒	☐	ChIP-seq
☒	☐	Flow cytometry
☒	☐	MRI-based neuroimaging

Plants

Seed stocks	pgr5CAS seeds (lacking the PGR5 protein) were obtained as a gift from Prof. Dario Leister and was described in Penzler et al. 2022. Seeds for pgr5 ndho were a gift form Eva-Marie Aro and described in Degen et al. 2023
Novel plant genotypes	pgr5CAS and pgr5 ndho were transformed using the floral dip method using Agrobacterium containing the TKTP-ATeam plasmid. Plants were selected using resistance to glufosinate ammonium and lines with high biosensors fluorescence were selected.
Authentication	Growth assays comparing untransformed with transformed WT, pgr5CAS and pgr5 ndho plants were performed, showing no detrimental effect on plant health.
