# Supplementary material for: Disequilibrium between chloroplast proton motive force and ATP levels in Arabidopsis
Source: Nat Plants. 2026 Aug 17;12(9):1679–87. doi: 10.1038/s41477-026-02374-w (PMC13585562; doi:10.1038/s41477-026-02374-w)
Supplement: Supplementary file 3 [file 41477_2026_2374_MOESM3_ESM.pdf]

nature portfolio

Peer Review File

Disequilibrium between chloroplast proton motive force and ATP levels in Arabidopsis

Corresponding Author: Professor Matthew Johnson

Version 0:

Reviewer comments:

Reviewer #1

(Comments for the Author)
In a dogma, linear electron transport cannot meet the ATP/NADPH production ratio (1.5) required by the Calvin-Benson cycle, and additional ATP is believed to be supplied by cyclic electron transport around PSI (CET). The authors of this manuscript analyzed MgATP2- levels in chloroplasts using the MgATP2- fluorescent biosensor ATeam1.03-nD/nA system. Although the pmf level was reduced to 50-75% of the WT in the pgr5CAS line, the MgATP2- level was unaffected. The authors conclude that PGR5-dependent CET is necessary to establish the full size of pmf, which is required for photoprotection rather than ATP synthesis.

This is excellent work concerning a very important topic. I generally agree with the authors' conclusion, but the discussion should be more precise in the quantitative evaluation of CET's contribution to ATP synthesis.

Specific comments
1) I believe that the ATP level is maintained in the chloroplasts of the PGR5Cas line. The authors argue that the reduced CO2 fixation activity observed in some studies depended on photodamage rather than an ATP shortage. It is possible that the Calvin-Benson cycle is mildly down-regulated to keep the ATP level stable in the chloroplasts. Is it possible to monitor the rate of CO2 fixation in the system?
2) In Fig. S2, a decline in pmf appears to be associated with low ATP levels.
3) The authors claim that the ATP/NADPH ratio is primarily balanced by exporting reducing equivalents from chloroplasts. I do not deny the possibility, but Y(II) is quantitatively consistent with the rate of CO2 fixation in many studies, suggesting that almost all reducing equivalents are used for CO2 fixation. The idea cannot fully explain the observation in this work.
4) The NDH complex might contribute more effectively to ATP synthesis through its proton-pumping activity. It could supplement PGR5 function in the mutant, especially given the low pmf. The manuscript underestimates its role.
5) The group of authors published a study demonstrating the role of PGR5-dependent CET in maintaining ATP levels under fluctuating conditions. This should be mentioned to thoroughly discuss the function of PGR5-dependent CET in a natural light environment.
6) Lines207-209. High luminal pH increases Q-cycle activity in the pgr5 mutant. Without electron donation to the PQ pool (or b6f), the Q cycle likely stops earlier in the pgr5 mutant than in the WT. These factors may influence the gH+ level.

Reviewer #2

(Comments for the Author)
Your results are intriguing, showing that a drop in proton-motive force doesn't translate into lower $MgATP^{2-}$ levels, which challenges the idea that PGR5-CET is the main ATP booster. Obviously, an interesting study; however, it requires significant improvement.
1) How was the ATeam1.03-nD/nA sensor calibrated in vivo, and were any controls performed to verify that changes in fluorescence truly reflect changes in $MgATP^{2-}$ rather than pH or ion-strength effects?
2) Was the pmf measured directly (e.g., by electrochromic shift or thylakoid membrane voltage probes), or inferred from indirect readouts? If indirect, could the discrepancy arise from differences in measurement sensitivity?
3) Did you monitor ATP consumption rates (e.g., by ^{32}P-labeling or ATPase assays) to rule out compensatory up-regulation of other ATP-producing pathways in the pgr5CAS background?
4) Were there any changes in the $NADPH/NADP^+$ ratio or in the activation state of Calvin-cycle enzymes that might offset the need for extra ATP?
5) Have you considered the possibility that the ATeam sensor is near saturation under your experimental conditions, so that modest changes in ATP are not resolved?
6) Since the results show that NPQ and pmf are low in the pgr5CAS mutant. Did you check the PSBS protein or gene

expression? Certainly, this data is important in showing the relationship between the NPQ and PMF.
7) Why is P700 oxidation more in pgr5CAS-TA1, and can you quantify the PsaA/B protein under such conditions?

Reviewer #3

(Comments for the Author)
In this manuscript, Degen and colleagues disentangled the contribution of PGR5 in the generation of a chloroplast proton motive force (pmf) and ATP synthesis in the chloroplast of Arabidopsis. During the last couple of decades, the scientific consensus for the PGR5-dependent cyclic electron flow was that it can generate an excess of pmf, which could translate into larger levels of ATP without the generation of NADPH. This could compensate for an imbalance in the ATP/NADPH ratio for the CBB cycle, under certain environmental conditions. The data shown by Degen et al challenge this consensus, demonstrating that, while mutating pgr5 disrupts the generation of a pmf, it does not impair ATP levels. Their work is experimentally sound, their hypothesis is clear, and several control experiments were performed to support the validity of their results. Overall, this is a well-written manuscript adding novel evidence on the role of PGR5 and cyclic electron transport in the regulation of photosynthesis. The following are some questions and suggestions to improve the manuscript.

1- I found it difficult to understand the way significant differences and statistics are shown in your figures. For instance, in Fig. 1f, based on the symbols, it seems that there are no significant differences between 21 and 113 µE at 2.5min, although the curves are clearly different. Same, for instance, in some cases in Fig. 2b and 2e. Maybe using * above the curves is clearer.
2- Supplementary Fig. 1d is important for the validity of your biosensor, since it confirms the dynamic range of ATP that it is capable of resolving. However, for this data (as well as for Fig. S1b), no statistical test was performed. Please include a statistical test to show that the ATP dynamic range is valid for performing your experiment.
3- When defining your experimental conditions in the confocal microscope, and to make them comparable with measurements performed in attached leaves you use NPQ as the main parameter (Fig. 1e and Fig. S1b). Although both parameters are related, what is the argument for choosing NPQ instead of qP (or, for instance, the quantum yield of PSII)? It seems more logical to compare the ATP generation with the electrons targeted to photochemistry instead of non-photochemical quenching.
4- It would be interesting to broaden the scope of your discussion in terms of how the ATP/NADPH ratio is balanced based on export of reducing equivalents to the cytosol via the malate valve. As you mentioned in paragraph L211, your data points to a larger relevance of this process in fulfilling the requirements of the CBB cycle. However, discussion related to how your data and the malate valve fit this model is a bit scarce.

Decision Letter:

17th December 2025

Dear Professor Johnson,

Your Brief Communication, "Disequilibrium between chloroplast proton motive force and ATP levels in Arabidopsis" has now been seen by three referees. You will see from their comments below that while they find your work of interest, some important points are raised. We are interested in the possibility of publishing your study in Nature Plants, but would like to consider your response to these concerns in the form of a revised manuscript before we make a final decision on publication.

As you can read below, all reviewers think that the findings are interesting and novel, however, some concerns are raised which should be addressed. In our opinion the main points are:

1. Both reviewers #1 and #2 are wondering whether the pgr5 mutant may lead to a downregulation of CBB cycle activity and hence obliterate the need for additional ATP production.
2. Reviewer #1 thinks that previous studies showing that Y(II) is quantitatively consistent with the rate of CO_2 fixation argue against the conclusion that ATP/NADPH ratio is primarily balanced by exporting reducing equivalents from chloroplasts. Similarly, reviewer #2 wonders whether there may be compensatory mechanisms for ATP production in the mutant. The editor wonders whether ATP may be imported through nucleotide transporters in pgr5.
3. Reviewer #3 thinks that statistical tests are required for the biosensor data showing the dynamic range of ATP, and it should be explained why ATP generation was compared to NPQ rather than qP or Y(II).

All referees also raise some other points that should be addressed. As usual, please do not hesitate to get in touch if you would like to discuss these issues further.

We therefore invite you to revise your manuscript taking into account all reviewer and editor comments. Please highlight all changes in the manuscript text file, preferably in Microsoft Word format.

We are committed to providing a fair and constructive peer-review process. Do not hesitate to contact us if there are specific requests from the reviewers that you believe are technically impossible or unlikely to yield a meaningful outcome.

When revising your manuscript please:

* Include a "Response to referees" document detailing, point-by-point, how you addressed each referee comment. If no action was taken to address a point, you must provide a compelling argument. This response will be sent back to the referees along

with the revised manuscript.

* If you have not done so already please begin to revise your manuscript so that it conforms to our Brief Communication format instructions at http://www.nature.com/nplants/info/final-submission. Refer also to any guidelines provided in this letter.

* Include a revised version of any required reporting checklist. It will be available to referees (and, potentially, statisticians) to aid in their evaluation if the manuscript goes back for peer review. A revised checklist is essential for re-review of the paper.

* Pay close attention to our href="https://www.nature.com/nature-portfolio/editorial-policies/image-integrity">Digital Image Integrity Guidelines. and to the following points. Please ensure:
-- that unprocessed scans are clearly labelled and match the gels and western blots presented in figures.
-- that control panels for gels and western blots are appropriately described as loading on sample processing controls
-- that all images in the paper are checked for duplication of panels and for splicing of gel lanes.
-- that you retain unprocessed data and metadata files after publication, ideally archiving data in perpetuity. These may be requested during the peer review and production process or after publication if any issues arise.

EXTENDED DATA FIGURES

When re-submitting your manuscript, please ensure that any supplementary figures and tables that are crucial to the manuscript's conclusions are converted into Extended Data figures and tables to increase visibility of these data. Extended Data figures and tables are online-only (present in the online PDF and full-text HTML versions of the paper), peer-reviewed display items that provide essential background to the article but are not included in the main article due to space constraints. A maximum of ten Extended Data display items (figures and tables) is permitted.

Nature Plants strongly supports public availability of data and are therefore keen that the data used in your paper is placed in an appropriate public data repository. Alternatively, if this is not possible, you may present the data as Extended Data or Supplementary Information. If data can only be shared on request, please explain why in your Data Availability Statement, and also in the correspondence with your editor. Please note that for some data types, deposition in a public repository is mandatory.

Please use the link below to submit your revised manuscript and related files:

Link Redacted

Note: This URL links to your confidential home page and associated information about manuscripts you may have submitted, or that you are reviewing for us. If you wish to forward this email to co-authors, please delete the link to your homepage.

We hope to receive your revised manuscript within three to four months, however, this is not a strict deadline. If you cannot send it within this time, please let us know. We will be happy to consider your revision so long as nothing similar has been accepted for publication at Nature Plants or published elsewhere.

We look forward to seeing the revised manuscript and thank you for the opportunity to review your work.

Sincerely,

Editor
Nature Plants

Nature Plants is committed to improving transparency in authorship. As part of our efforts in this direction, we are now requesting that all authors identified as 'corresponding author' on published papers create and link their Open Researcher and Contributor Identifier (ORCID) with their account on the Manuscript Tracking System (MTS), prior to acceptance. This applies to primary research papers only. ORCID helps the scientific community achieve unambiguous attribution of all scholarly contributions. You can create and link your ORCID from the home page of the MTS by clicking on 'Modify my Springer Nature account'. For more information please visit please visit www.springernature.com/orcid.

Please do not hesitate to contact me if you have any questions or would like to discuss these revisions further.

****

Reviewers' Comments:

Reviewer #1 (Comments for the Author):

In a dogma, linear electron transport cannot meet the ATP/NADPH production ratio (1.5) required by the Calvin-Benson cycle, and additional ATP is believed to be supplied by cyclic electron transport around PSI (CET). The authors of this manuscript analyzed $MgATP^{2-}$ levels in chloroplasts using the $MgATP^{2-}$ fluorescent biosensor ATeam1.03-nD/nA system. Although the

pmf level was reduced to 50-75% of the WT in the pgr5CAS line, the $MgATP^{2-}$ level was unaffected. The authors conclude that PGR5-dependent CET is necessary to establish the full size of pmf, which is required for photoprotection rather than ATP synthesis.

This is excellent work concerning a very important topic. I generally agree with the authors' conclusion, but the discussion should be more precise in the quantitative evaluation of CET's contribution to ATP synthesis.

Specific comments
1) I believe that the ATP level is maintained in the chloroplasts of the PGR5Cas line. The authors argue that the reduced CO_2 fixation activity observed in some studies depended on photodamage rather than an ATP shortage. It is possible that the Calvin-Benson cycle is mildly down-regulated to keep the ATP level stable in the chloroplasts. Is it possible to monitor the rate of CO_2 fixation in the system?
2) In Fig. S2, a decline in pmf appears to be associated with low ATP levels.
3) The authors claim that the ATP/NADPH ratio is primarily balanced by exporting reducing equivalents from chloroplasts. I do not deny the possibility, but Y(II) is quantitatively consistent with the rate of CO_2 fixation in many studies, suggesting that almost all reducing equivalents are used for CO_2 fixation. The idea cannot fully explain the observation in this work.
4) The NDH complex might contribute more effectively to ATP synthesis through its proton-pumping activity. It could supplement PGR5 function in the mutant, especially given the low pmf. The manuscript underestimates its role.
5) The group of authors published a study demonstrating the role of PGR5-dependent CET in maintaining ATP levels under fluctuating conditions. This should be mentioned to thoroughly discuss the function of PGR5-dependent CET in a natural light environment.
6) Lines207-209. High luminal pH increases Q-cycle activity in the pgr5 mutant. Without electron donation to the PQ pool (or b6f), the Q cycle likely stops earlier in the pgr5 mutant than in the WT. These factors may influence the gH^+ level.

Reviewer #2 (Comments for the Author):

Your results are intriguing, showing that a drop in proton-motive force doesn't translate into lower $MgATP^{2-}$ levels, which challenges the idea that PGR5-CET is the main ATP booster. Obviously, an interesting study; however, it requires significant improvement.
1) How was the ATeam1.03-nD/nA sensor calibrated in vivo, and were any controls performed to verify that changes in fluorescence truly reflect changes in $MgATP^{2-}$ rather than pH or ion-strength effects?
2) Was the pmf measured directly (e.g., by electrochromic shift or thylakoid membrane voltage probes), or inferred from indirect readouts? If indirect, could the discrepancy arise from differences in measurement sensitivity?
3) Did you monitor ATP consumption rates (e.g., by ^{32}P-labeling or ATPase assays) to rule out compensatory up-regulation of other ATP-producing pathways in the pgr5CAS background?
4) Were there any changes in the $NADPH/NADP^+$ ratio or in the activation state of Calvin-cycle enzymes that might offset the need for extra ATP?
5) Have you considered the possibility that the ATeam sensor is near saturation under your experimental conditions, so that modest changes in ATP are not resolved?
6) Since the results show that NPQ and pmf are low in the pgr5CAS mutant. Did you check the PSBS protein or gene expression? Certainly, this data is important in showing the relationship between the NPQ and PMF.
7) Why is P700 oxidation more in pgr5CAS-TA1, and can you quantify the PsaA/B protein under such conditions?

Reviewer #3 (Comments for the Author):

In this manuscript, Degen and colleagues disentangled the contribution of PGR5 in the generation of a chloroplast proton motive force (pmf) and ATP synthesis in the chloroplast of Arabidopsis. During the last couple of decades, the scientific consensus for the PGR5-dependent cyclic electron flow was that it can generate an excess of pmf, which could translate into larger levels of ATP without the generation of NADPH. This could compensate for an imbalance in the ATP/NADPH ratio for the CBB cycle, under certain environmental conditions. The data shown by Degen et al challenge this consensus, demonstrating that, while mutating pgr5 disrupts the generation of a pmf, it does not impair ATP levels. Their work is experimentally sound, their hypothesis is clear, and several control experiments were performed to support the validity of their results. Overall, this is a well-written manuscript adding novel evidence on the role of PGR5 and cyclic electron transport in the regulation of photosynthesis. The following are some questions and suggestions to improve the manuscript.

1- I found it difficult to understand the way significant differences and statistics are shown in your figures. For instance, in Fig. 1f, based on the symbols, it seems that there are no significant differences between 21 and 113 µE at 2.5min, although the curves are clearly different. Same, for instance, in some cases in Fig. 2b and 2e. Maybe using * above the curves is clearer.
2- Supplementary Fig. 1d is important for the validity of your biosensor, since it confirms the dynamic range of ATP that it is capable of resolving. However, for this data (as well as for Fig. S1b), no statistical test was performed. Please include a statistical test to show that the ATP dynamic range is valid for performing your experiment.
3- When defining your experimental conditions in the confocal microscope, and to make them comparable with measurements performed in attached leaves you use NPQ as the main parameter (Fig. 1e and Fig. S1b). Although both parameters are related, what is the argument for choosing NPQ instead of qP (or, for instance, the quantum yield of PSII)? It seems more logical to compare the ATP generation with the electrons targeted to photochemistry instead of non-photochemical quenching.

4- It would be interesting to broaden the scope of your discussion in terms of how the ATP/NADPH ratio is balanced based on export of reducing equivalents to the cytosol via the malate valve. As you mentioned in paragraph L211, your data points to a larger relevance of this process in fulfilling the requirements of the CBB cycle. However, discussion related to how your data and the malate valve fit this model is a bit scarce.

********************END********************

Version 1:

Reviewer comments:

Reviewer #1

(Comments for the Author)
The authors addressed all of my concerns, and I was largely satisfied with the revision. I still have a comment on the evaluation of the pgr5CAS ndhO double mutant, and I would be happy if they considered the point before publication.
1) The double mutant is very sick, and the low activity of PSI likely limits CO_2 fixation rather than ATP level (Kobayashi et al. Plant J 2024). I guess that the levels of the Calvin cycle enzymes were also reduced, contributing to the balance of light and CO_2 fixation reactions. Even in the NDH-less single mutant, CO_2 fixation was reduced in low light in rice. I do not request you to analyze the single mutant. I fully agree with the authors that it is a key to downregulate ATP synthase activity to preserve the size of pmf (P5, lines 40-44). However, I still believe the CET contributes to ATP synthesis on some occasions. I look forward to seeing the genetic study that examines the impact of blocking the transfer of reducing power from the stroma to the cytosol in the future.
2) P2, line 3. NAD(P)H-like dehydrogenase should be NADH dehydrogenase-like. I prefer to use NADH because the core of the complex is similar to that of respiratory complex I.

Toshihary Shikanai

Reviewer #2

(Comments for the Author)
The authors have satisfactorly answered all queries and also, after the revision the manuscript has been improved.

Reviewer #3

(Comments for the Author)
The authors have sufficiently revised and improved their manuscript.

Decision Letter:

Our ref: NPLANTS-251121414A

3rd June 2026

Dear Dr. Johnson,

Thank you for submitting your revised manuscript "Disequilibrium between chloroplast proton motive force and ATP levels in Arabidopsis" (NPLANTS-251121414A). It has now been seen by the original referees and their comments are below. The reviewers find that the paper has improved in revision, and therefore we'll be happy in principle to publish it in Nature Plants, pending minor revisions to satisfy the referees' final requests and to comply with our editorial and formatting guidelines.

If the current version of your manuscript is in a PDF format, please email us a copy of the file in an editable format (Microsoft Word or LaTex)-- we can not proceed with PDFs at this stage.

We are now performing detailed checks on your paper and will send you a checklist detailing our editorial and formatting requirements in about a week. Please do not upload the final materials and make any revisions until you receive this additional information from us.

Thank you again for your interest in Nature Plants Please do not hesitate to contact me if you have any questions.

Sincerely,

Editor
Nature Plants

Reviewer #1 (Comments for the Author):

The authors addressed all of my concerns, and I was largely satisfied with the revision. I still have a comment on the evaluation of the pgr5CAS ndhO double mutant, and I would be happy if they considered the point before publication.
1) The double mutant is very sick, and the low activity of PSI likely limits CO_2 fixation rather than ATP level (Kobayashi et al. Plant J 2024). I guess that the levels of the Calvin cycle enzymes were also reduced, contributing to the balance of light and CO_2 fixation reactions. Even in the NDH-less single mutant, CO_2 fixation was reduced in low light in rice. I do not request you to analyze the single mutant. I fully agree with the authors that it is a key to downregulate ATP synthase activity to preserve the size of pmf (P5, lines 40-44). However, I still believe the CET contributes to ATP synthesis on some occasions. I look forward to seeing the genetic study that examines the impact of blocking the transfer of reducing power from the stroma to the cytosol in the future.
2) P2, line 3. NAD(P)H-like dehydrogenase should be NADH dehydrogenase-like. I prefer to use NADH because the core of the complex is similar to that of respiratory complex I.

Toshihary Shikanai

Reviewer #2 (Comments for the Author):

The authors have satisfactorly answered all queries and also, after the revision the manuscript has been improved.

Reviewer #3 (Comments for the Author):

The authors have sufficiently revised and improved their manuscript.

Version 2:

Decision Letter:

17th July 2026

Dear Professor Johnson,

We are pleased to inform you that your Brief Communication entitled "Disequilibrium between chloroplast proton motive force and ATP levels in Arabidopsis", has now been accepted for publication in Nature Plants.

Over the next few weeks, your paper will be copyedited to ensure that it conforms to Nature Plants style. We look particularly carefully at the titles of all papers to ensure that they are relatively brief and understandable.

Once your paper is typeset, you will receive an email with a link to choose the appropriate publishing options for your paper and our Author Services team will be in touch regarding any additional information that may be required.

After the grant of rights is completed, you will receive a link to your electronic proof via email with a request to make any corrections within 48 hours. If, when you receive your proof, you cannot meet this deadline, please inform us at rjsproduction@springernature.com immediately.

You will not receive your proofs until the publishing agreement has been received through our system.

You may wish to make your media relations office aware of your accepted publication, in case they consider it appropriate to organize some internal or external publicity. Once your paper has been scheduled you will receive an email confirming the publication details. This is normally 3-4 working days in advance of publication. If you need additional notice of the date and time of publication, please let the production team know when you receive the proof of your article to ensure there is sufficient time to coordinate. Further information on our embargo policies can be found here:
https://www.nature.com/authors/policies/embargo.html

Due to the importance of these deadlines, we ask you please us know now whether you will be difficult to contact over the next month. If this is the case, we ask you provide us with the contact information (email, phone and fax) of someone who will be able to check the proofs on your behalf, and who will be available to address any last-minute problems.

Acceptance of your manuscript is conditional on all authors' agreement with our publication policies (see http://www.nature.com/authors/policies/index.html). In particular your manuscript must not be published elsewhere.

If you have any questions about our publishing options, costs, Open Access requirements, or our legal forms, please contact ASJournals@springernature.com

Authors may need to take specific actions to achieve compliance with funder and institutional open access mandates. If your research is supported by a funder that requires immediate open access (e.g. according to Plan S principles or the NIH public access policy) then you should select the gold OA route, and we will direct you to the compliant route where possible. Because authors warrant under our subscription licensing terms that they haven't committed to licensing any version of their article under a licence inconsistent with the terms of our agreement – including the applicable embargo period – publication under the subscription model isn't suitable for authors whose funders require no embargo.

An online order form for reprints of your paper is available at https://www.nature.com/reprints/author-reprints.html. All co-authors, authors' institutions and authors' funding agencies can order reprints using the form appropriate to their geographical region.

We welcome the submission of potential cover material (including a short caption of around 40 words) related to your manuscript; suggestions should be sent to Nature Plants as electronic files (the image should be 300 dpi at 210 x 297 mm in either TIFF or JPEG format). Please note that such pictures should be selected more for their aesthetic appeal than for their scientific content, and that colour images work better than black and white or grayscale images. Please include a written description of your image and how it was created along with your image files. Please do not try to design a cover with the Nature Plants logo etc., and please do not submit composites of images related to your work. I am sure you will understand that we cannot make any promise as to whether any of your suggestions might be selected for the cover of the journal.

You can now use a single sign-on for all your accounts, view the status of all your manuscript submissions and reviews, access usage statistics for your published articles and download a record of your refereeing activity for the Nature journals.

To assist our authors in disseminating their research to the broader community, our SharedIt initiative provides you with a unique shareable link that will allow anyone (with or without a subscription) to read the published article. Recipients of the link with a subscription will also be able to download and print the PDF.

As soon as your article is published, you will receive an automated email with your shareable link.

With kind regards,

Editor
Nature Plants

P.S. Click on the following link if you would like to recommend Nature Plants to your librarian http://www.nature.com/subscriptions/recommend.html#forms

** Visit the Springer Nature Editorial and Publishing website at www.springernature.com/editorial-and-publishing-jobs for more information about our career opportunities. If you have any questions please click here.**

Open Access This Peer Review File is licensed under a Creative Commons Attribution 4.0 International License, which permits use, sharing, adaptation, distribution and reproduction in any medium or format, as long as you give appropriate credit to the original author(s) and the source, provide a link to the Creative Commons license, and indicate if changes were made. In cases where reviewers are anonymous, credit should be given to 'Anonymous Referee' and the source.
The images or other third party material in this Peer Review File are included in the article's Creative Commons license, unless indicated otherwise in a credit line to the material. If material is not included in the article's Creative Commons license and your intended use is not permitted by statutory regulation or exceeds the permitted use, you will need to obtain permission directly from the copyright holder.
To view a copy of this license, visit https://creativecommons.org/licenses/by/4.0/

Reviewers' Comments:

Reviewer #1 (Comments for the Author):

In a dogma, linear electron transport cannot meet the ATP/NADPH production ratio (1.5) required by the Calvin-Benson cycle, and additional ATP is believed to be supplied by cyclic electron transport around PSI (CET). The authors of this manuscript analyzed MgATP2- levels in chloroplasts using the MgATP2- fluorescent biosensor ATeam1.03-nD/nA system. Although the pmf level was reduced to 50-75% of the WT in the pgr5CAS line, the MgATP2- level was unaffected. The authors conclude that PGR5-dependent CET is necessary to establish the full size of pmf, which is required for photoprotection rather than ATP synthesis.

This is excellent work concerning a very important topic. I generally agree with the authors' conclusion, but the discussion should be more precise in the quantitative evaluation of CET's contribution to ATP synthesis.

Specific comments:

1) I believe that the ATP level is maintained in the chloroplasts of the PGR5Cas line. The authors argue that the reduced CO2 fixation activity observed in some studies depended on photodamage rather than an ATP shortage. It is possible that the Calvin-Benson cycle is mildly down-regulated to keep the ATP level stable in the chloroplasts. Is it possible to monitor the rate of CO2 fixation in the system?

- As a direct *in vivo* readout of CBB-driven ATP consumption within the same system, we draw the reviewer's attention to the rate of stromal $MgATP^{2-}$ decline in the dark immediately following illumination. This post-illumination decay reflects continued ATP turnover by the CBB cycle once photophosphorylation has ceased, and it is comparable between WT-TA and *pgr5CAS*-TA1 at matched light intensities (Fig 1e and f). The rate of CBB-driven ATP consumption is therefore not appreciably decreased in *pgr5CAS* under our conditions, so the maintained $MgATP^{2-}$ level cannot be explained by a compensatory fall in ATP demand since this would slow the rate of consumption in the dark relative to the WT. This directly addresses the reviewer's concern without requiring additional gas-exchange measurements.
- We note that the dark-decay measurement was performed under saturating CO_2 ; the ΔpH-attenuation model predicts that under ambient CO_2 , the regime in which the published *pgr5* fixation deficit is most pronounced, a modest slowing of the post-illumination decay would be expected in *pgr5CAS* relative to WT, and we now identify this as a testable prediction for follow-up work.
- This is consistent with past work showing Redox activation of CCB enzymes is WT-like in *pgr5* according to several studies. Okegawa etc al., 2022 Plant Cell Physiol. 63; 92-103 (2022); Okegawa et al., Plant Cell 32; 3866-3883 (2020)

2) In Fig. S2, a decline in pmf appears to be associated with low ATP levels.

- We rechecked this, firstly there are no statistically significant differences in ATP levels observed between WT and *pgr5* in the original Figure S2e, now renamed Supplementary Figure S1e. In line with other publications using the ATP biosensor, we replotted this and all other data in our manuscript by normalising the FRET ratio to the end of the dark period (time =0) followed by log10 transformation (see Col et al., eLife 6, 4378 (2017) and Scherschel et al., Nature Comm. 15, 10704 (2024).

.

3) The authors claim that the ATP/NADPH ratio is primarily balanced by exporting reducing equivalents from chloroplasts. I do not deny the possibility, but Y(II) is quantitatively consistent with the rate of CO_2 fixation in many studies, suggesting that almost all reducing equivalents are used for CO_2 fixation. The idea cannot fully explain the observation in this work.

- Here we would highlight the recent cytosolic $NADH/NAD^+$ biosensor data in our preprint by Zheng et al., 2025 that shows a fast and pronounced reduction of the cytosolic NAD^+ pool upon illumination. This clearly shows that reductant export occurs rapidly and efficiently. We more carefully discuss these data in the revised manuscript in comparison with classical work by Genty et al., 1989.

4) The NDH complex might contribute more effectively to ATP synthesis through its proton-pumping activity. It could supplement PGR5 function in the mutant, especially given the low pmf. The manuscript underestimates its role.

- We thank the reviewer for this comment. This idea provoked us to measure the *pgr5 ndho* double mutant which lacks both CET pathways, once again there is no significant effect on chloroplast ATP levels (see Figure 3). This underlines our central finding that considerable disequilibrium exists between pmf and [$MgATP^{2-}$] and moreover that CET's main function cannot be ATP augmentation.

5) The group of authors published a study demonstrating the role of PGR5-dependent CET in maintaining ATP levels under fluctuating conditions. This should be mentioned to thoroughly discuss the function of PGR5-dependent CET in a natural light environment.

- The reviewer is correct to say we suggested perturbed ATP levels in *pgr5* lead to photosynthetic oscillations (Degen et al., JXB, 75, 947-961 (2024)). However, we had no direct evidence for ATP shortfall in this study. Here, we revise our previous hypothesis based on direct measurements. We now more carefully discuss these points in the revised manuscript and more clearly refer to our previous work.

6) Lines207-209. High luminal pH increases Q-cycle activity in the pgr5 mutant. Without electron donation to the PQ pool (or b6f), the Q cycle likely stops earlier in the pgr5 mutant than in the WT. These factors may influence the gH+ level.

- Yes this is a possible cause of high gH+ in pgr5 as previously suggested by Yamamoto and Shikanai Plant Physiol. 184, 421–427 (2020). We now reference this paper in the revised discussion.

Reviewer #2 (Comments for the Author):

Your results are intriguing, showing that a drop in proton- motive force doesn't translate into lower $MgATP^{2-}$ levels, which challenges the idea that PGR5- CET is the main ATP booster. Obviously, an interesting study; however, it requires significant improvement.

1) How was the ATeam1.03- nD/nA sensor calibrated in vivo, and were any controls performed to verify that changes in fluorescence truly reflect changes in $MgATP^{2-}$ rather than pH or ion- strength effects?

- Calibration was performed *in vivo* using digitonin to allow ATP infiltration into the leaves (see Supplementary Figure 3f). The suggested concentration of ATP in the chloroplast stroma is reported in the range 0.5 to 1.8 mM (Refs 19-22). Our infiltration data show the ATeam1.03- nD/nA sensor responds to the difference between 1.6 mM and 3.2 mM ATP proportionally.
- We have previously tested different ions when characterizing the ATeam sensor *in vitro* (Col et al., eLife 6, 4378 (2017). None had an effect, except for Mg^{2+} when it was limiting for the $MgATP^{2-}$ complex to form. But since chloroplast Mg^{2+} levels exceed those of ATP (estimated at 2-10 mM by IshIjima et al., Arch Biochem Biophys, 2003 and Portis et al., 1976, Plant Physiol. versus 0.5-1.8 mM for ATP (Refs 19-22) this is unlikely to be a factor in our experiments).
- Past work has shown that ATeam1.03- nD/nA is insensitive to pH from pH 7.1 to 8.5, the expected range for the chloroplast stroma is pH 7-8 (Refs 27, 28). In terms of the pKa values, mVenus has a pKa of ~6.0, meaning fluorescence drops off significantly only below pH 6, with the transition centred around that value (Col et al., eLife 6, 4378 (2017)). Indeed, even if lower ΔpH in *pgr5CAS* translated into a more acidic stroma compared to WT, since YFP (and its Venus variant used in ATeam) becomes *less* fluorescent at low pH i.e. is acid-quenched, were this pH sensitivity a significant factor this should tend to *decrease* the apparent $MgATP^{2-}$ level in *pgr5CAS* (something that is clearly not observed see Figure 1) rather than artificially increase it.
- Moreover, we also find that changes in the biosensor readout are responsive to DCMU which inhibits photosynthetic electron transfer in chloroplasts and indeed inhibits the fluorescence rise associated with $MgATP^{2-}$ formation (Figure 1). This is backed up by past work (see Voon, C. P. et al. (2018)) with a range of inhibitors.
- Finally, we now include an additional control of oligomycin, which inhibits the mitochondrial ATP synthase (see Supplementary Figure 5) but does not inhibit the chloroplast ATP synthase (see Krömer et al., FEBS Letts, 226, 352-356 (1988) and Krömer and Heldt Plant Physiol., 95, 1270–1276 (1991)). This data shows both *pgr5CAS* and WT plants can significantly increase their chloroplast ATP levels when treated with oligomycin, likely due to the increased availability of inorganic phosphate

as cytosolic ATP levels decline. This confirms that the amount of chloroplast $MgATP^{2-}$ observed in either set of biosensor transformed plants does not represent an artificial ceiling of sensitivity.

2) Was the pmf measured directly (e.g., by electrochromic shift or thylakoid membrane voltage probes), or inferred from indirect readouts? If indirect, could the discrepancy arise from differences in measurement sensitivity?

- Directly via ECS method, see Figure 2. This is the current standard in the field. Complimentary work using 9-aminocaridine fluorescence on isolated chloroplasts in *pgr5* versus WT support the differences observed via ECS e.g. Yamamoto and Shikanai Plant Physiol. 184, 421–427 (2020).

3) Did you monitor ATP consumption rates (e.g., by ^{32}P- labeling or ATPase assays) to rule out compensatory up- regulation of other ATP- producing pathways in the pgr5CAS background?

- As a direct *in vivo* readout of CBB-driven ATP consumption within the same system, we draw the reviewer's attention to the rate of stromal $MgATP^{2-}$ decline in the dark immediately following illumination. This post-illumination decay reflects continued ATP turnover by the CBB cycle once photophosphorylation has ceased, and it is comparable between WT-TA and *pgr5CAS*-TA1 at matched light intensities (Fig 1e-f). The rate of CBB-driven ATP consumption is therefore not appreciably decreased in *pgr5CAS* under our conditions, so the maintained $MgATP^{2-}$ level cannot be explained by a compensatory fall in ATP demand since this would slow the rate of consumption in the dark relative to the WT. This directly addresses the reviewer's concern without requiring additional ^{32}P- labeling or ATPase assays.
- We note that the dark-decay measurement was performed under saturating CO_2 ; the ΔpH-attenuation model predicts that under ambient CO_2 , the regime in which the published *pgr5* fixation deficit is most pronounced, a modest slowing of the post-illumination decay would be expected in *pgr5CAS* relative to WT, and we now identify this as a testable prediction for follow-up work.
- We also tested whether mitochondrial ATP production might make up for any decrease in photosynthetic ET derived ATP production in *pgr5* by infiltrating leaves with the mitochondrial ATP synthase inhibitor oligomycin (Supplementary Figure 5 and answer to R2 Point 1). The data show that oligomycin treatment actually *increases* chloroplast ATP levels in both *pgr5CAS* and WT proportionally. This confirms both that our sensor has additional dynamic range over that seen in Figure 1 and moreover that mitochondrial derived ATP does not supplement ATP levels in *pgr5*.

4) Were there any changes in the $NADPH/NADP^+$ ratio or in the activation state of Calvin- cycle enzymes that might offset the need for extra ATP?

- Redox activation of CCB enzymes is WT-like in *pgr5* according to several studies. Okegawa et al., 2022 Plant Cell Physiol. 63; 92-103 (2022); Okegawa et al., Plant Cell 32; 3866-3883 (2020).
- Changes in $NADPH/NADP^+$ ratio in *pgr5CAS* is the source of a future work using our new NapStar biosensor (Scherschel et al., 2025, Nat Comms). A full NapStar analysis is beyond the scope of this Brief Communication and forms the basis of a follow-up study, but we note that since Fd pool and PSI acceptors are over-reduced in *pgr5* that logically the NADPH/NADP+ pool would be more reduced and thus NADPH is unlikely to be limiting to CO2 fixation in this mutant

5) Have you considered the possibility that the ATeam sensor is near saturation under your experimental conditions, so that modest changes in ATP are not resolved?

- This is a critical point which we have been mindful about. As discussed above the oligomycin data and the infiltration data both demonstrate that the upper limit was not reached in the measurements (see answers to R2 Point 1).

6) Since the results show that NPQ and pmf are low in the pgr5CAS mutant. Did you check the PSBS protein or gene expression? Certainly, this data is important in showing the relationship between the NPQ and PMF.

- Past work has shown that PsbS protein levels are WT-like in *pgr5* (see e.g. Degen et al., Plant Physiol. 192, 370–386 (2023)

7) Why is P700 oxidation more in pgr5CAS-TA1, and can you quantify the PsaA/B protein under such conditions?

- Consistent with previous publications (Okegawa et al. 2007, Rühle et al. 2021, Degen et al, 2022) P700 oxidation is faster in *pgr5* mutants (and conversely slower in high CET mutants like *hope2*) because re-reduction of PSI via cyclic electron transfer is abolished, thus PSI oxidises faster. Past work has shown that PsaA/B levels fall in pgr5 in fluctuating light consistent with PSI photoinhibition but we do not revisit this work here (Soursa et al., 2012. Plant Cell).

Reviewer #3 (Comments for the Author):

In this manuscript, Degen and colleagues disentangled the contribution of PGR5 in the generation of a chloroplast proton motive force (pmf) and ATP synthesis in the chloroplast of Arabidopsis. During the last couple of decades, the scientific consensus for the PGR5-dependent cyclic electron flow was that it can generate an excess of pmf, which could translate into larger levels of ATP without the generation of NADPH. This could compensate for an imbalance in the ATP/NADPH ratio for the CBB cycle, under certain environmental conditions. The data shown by Degen et al challenge this consensus, demonstrating that, while mutating pgr5 disrupts the generation of a pmf, it does not impair ATP levels. Their

work is experimentally sound, their hypothesis is clear, and several control experiments were performed to support the validity of their results. Overall, this is a well-written manuscript adding novel evidence on the role of PGR5 and cyclic electron transport in the regulation of photosynthesis. The following are some questions and suggestions to improve the manuscript.

1- I found it difficult to understand the way significant differences and statistics are shown in your figures. For instance, in Fig. 1f, based on the symbols, it seems that there are no significant differences between 21 and 113 µE at 2.5min, although the curves are clearly different. Same, for instance, in some cases in Fig. 2b and 2e. Maybe using * above the curves is clearer.

- Thank you for the suggestion. We have now replaced different symbols with a * to show significance between WT and *pgr5CAS* or between treatments, except in some instances where usage of letters was preferred. This is always indicated in the figure legend.

2- Supplementary Fig. 1d is important for the validity of your biosensor, since it confirms the dynamic range of ATP that it is capable of resolving. However, for this data (as well as for Fig. S1b), no statistical test was performed. Please include a statistical test to show that the ATP dynamic range is valid for performing your experiment.

- We have performed a statistical test to show a significant difference between 0 mM, 1.6 mM and 3.2 mM ATP/$MgCl_2$ shown in Supplementary Figure 3f.

3- When defining your experimental conditions in the confocal microscope, and to make them comparable with measurements performed in attached leaves you use NPQ as the main parameter (Fig. 1e and Fig. S1b). Although both parameters are related, what is the argument for choosing NPQ instead of qP (or, for instance, the quantum yield of PSII)? It seems more logical to compare the ATP generation with the electrons targeted to photochemistry instead of non-photochemical quenching.

- We now include Y(II) and Y(NA) data in Supplementary Figure 3 b-d to show the comparisons between attached and detached leaves. 100 mM bicarbonate gives consistently the best results. Including Y(NA) also highlights that when only adding water to the detached leaves, PSI suffers from acceptor-side limitation, which is alleviated by including bicarbonate as a CO_2 source, providing an effective e^- sink.

4- It would be interesting to broaden the scope of your discussion in terms of how the ATP/NADPH ratio is balanced based on export of reducing equivalents to the cytosol via the malate valve. As you mentioned in paragraph L211, your data points to a larger relevance of this process in fulfilling the requirements of the CBB cycle. However, discussion related to how your data and the malate valve fit this model is a bit scarce.

- Thanks for this suggestion we have now added additional detail to this section of the discussion. See also answer to R1 Point 3.

Reviewers' Comments:

Reviewer #1 (Comments for the Author):

The authors addressed all of my concerns, and I was largely satisfied with the revision. I still have a comment on the evaluation of the pgr5CAS ndhO double mutant, and I would be happy if they considered the point before publication.
1) The double mutant is very sick, and the low activity of PSI likely limits CO_2 fixation rather than ATP level (Kobayashi et al. Plant J 2024). I guess that the levels of the Calvin cycle enzymes were also reduced, contributing to the balance of light and CO_2 fixation reactions. Even in the NDH-less single mutant, CO_2 fixation was reduced in low light in rice. I do not request you to analyze the single mutant. I fully agree with the authors that it is a key to downregulate ATP synthase activity to preserve the size of pmf (P5, lines 40-44). However, I still believe the CET contributes to ATP synthesis on some occasions. I look forward to seeing the genetic study that examines the impact of blocking the transfer of reducing power from the stroma to the cytosol in the future.

- Yes this is an excellent point and as the reviewer says we can test this in the future with further experimentation.

2) P2, line 3. NAD(P)H-like dehydrogenase should be NADH dehydrogenase-like. I prefer to use NADH because the core of the complex is similar to that of respiratory complex I.

- Thanks, we have made the requested change.

Toshihary Shikanai

Reviewer #2 (Comments for the Author):

The authors have satisfactorily answered all queries and also, after the revision the manuscript has been improved.

Reviewer #3 (Comments for the Author):

The authors have sufficiently revised and improved their manuscript.
